# Entropy-Driven Ligand Exchange in a Rotationally Flexible Dinuclear Fe(II)–Fe(II) Complex

**DOI:** 10.1021/acs.inorgchem.5c05481

**Published:** 2026-06-17

**Authors:** Pablo G. Porta, Benjamin Kintzel, Birgit Weber, Michael Busch, Dieter Sorsche

**Affiliations:** † Institute of Inorganic Chemistry I, 8125 Ulm University, Albert-Einstein-Allee 11, Ulm 89081, Germany; ‡ Institut für Anorganische und Analytische Chemie, 9378 Friedrich-Schiller-Universität Jena, Humboldtstraße 8, Jena 07743, Germany; § Department of Engineering Sciences and Mathematics, Applied Physics, 26523 Luleå University of Technology, Luleå 971 87, Sweden; ∥ Wallenberg Initiative Materials Science for Sustainability (WISE), 5185 Luleå University of Technology, Luleå 971 87, Sweden

## Abstract

In metalloenzymes, precise control of metal–metal distance and coordination environment enables challenging catalytic transformations. This often involves hydrogen bonding in the second coordination sphere. Although many homogeneous systems aim to mimic these features, the capture and characterization of transient coordination events remains a challenge. Exploring the first di-iron complex of the rotationally flexible dinucleating ligand 1,1′,5,5′,6,6′-hexamethyl-4,4′-bis­(picolinimino)-2,2′-bibenzimidazole, report an unexpected, entropy-driven ligand-exchange equilibrium which we describe by means of variable temperature studies in solution, key species fully structurally characterized *in crystallo*, and a mechanistic study *in silico*. This comprehensive experimental and computational characterization of the ligand and complex revealed how the central C–C bond enables adaptation to different metal–metal distances. Cooling a methanol solution of the complex induces a color change from green to blue which is attributed to the reversible substitution of chlorido ligands by methanol. Density functional theory calculations suggest that this ligand exchange is driven by the change in entropy inherent to the reduction of temperature. Under controlled conditions, electrochemical analysis reveals two accessible redox events: a fully reversible redox process at −0.05 V vs Fc/Fc^+^ and a series of irreversible reduction reactions at more negative potentials. The resemblance of these redox features with those in established iron catalysts highlights the potential of this system to support catalytic transformations under certain conditions.

## Graphical abstract

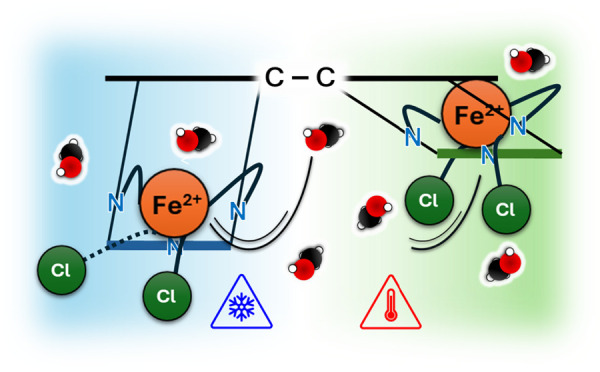

## Introduction

Dinuclear metal complexes, particularly those featuring iron centers, have garnered significant attention in inorganic chemistry due to their ability to model and mimic the cooperative reactivity of di- and oligonuclear iron enzymes, such as methane monooxygenase, [FeFe] hydrogenases, or the FeMo cofactor of nitrogenase.^1–7^ In order to enable cooperativity, the complexes are constructed by embedding iron into dinucleating ligands and placing two metal centers in close spatial proximity.^8–10^ Cooperativity between metal centers is generally predetermined by the geometry of the dinucleating ligand and consequently the distance between the two metal centers.^10–14^ This is contrasted by the self-assembly of dinuclear complexes from mononuclear precursors which provides more freedom in terms of relative orientation. An intermediate situation which combines the advantages of both approaches by allowing for the flexibility of self-assembled monomers while at the same time enforcing a degree of preassembly can be achieved by specifically tailored bridging ligands. Such ligands are rare, yet one notable example are alkylene-tethered bis­(pyridine-dimine) (PDI) ligands pioneered by Tomson and co-workers.^15–18^ However, the different orientations of the two metal centers relative to each other found within this series of complexes indicate low selectivity which is a disadvantage when trying to model the active centers of di-iron enzymes where the relative orientation of metal centers is predetermined by the protein framework.

A different design approach was previously developed by Muller, Bernardinelli, and Reedijk who reported the dinucleating ligand 1,1′,5,5′,6,6′-hexamethyl-4,4′-bis­(picolinimino)-2,2′-bibenzimidazole (^**Me**^ **bpbbi**) and demonstrated its ability to form dinuclear complexes of copper as potential models for the oxygen transport protein hemocyanine.^19^ The unique feature of the ^**Me**^ **bpbbi** ligand is its central C–C single bond which enables a rotational motion that directly correlates to the distance between the two metal centers while constraining their relative orientation toward each other. However, the ability of this ligand to vary the extent of direct interactions between the two metal centers through changing the metals’ relative orientation has never been fully explored. This is in part due to the susceptibility of this ligand to hydrolysis in the aqueous medium.

In this study,^20^ we report the first example of a diferrous complex, namely, {Fe_2_}, supported by the ^**Me**^ **bpbbi** ligand and describe its speciation in methanol by means of a stepwise ligand-exchange process leading to a counterintuitive increase in solubility upon cooling due to the entropy-driven ion pair formation. To this end, solid-state structures obtained from different solvent/nonsolvent combinations at different temperatures provided remarkably detailed insights. The dinucleating ligand plays a central role, as its rotational flexibility allows for different metal–metal distances depending on the ligand environment and, in one instance, enables the formation of an intramolecular hydrogen bond between the two ligand spheres (Figure 1). These structural insights are complemented by density functional theory (DFT) calculations, which map the ligand-exchange pathway and suggest that the preference for methanol coordination at low temperatures is driven by an entropy increase due to chloride dissociation. Further, bulk magnetometric measurements reflect a change from coordination number 5 to a more symmetric pseudooctahedral coordination as the corresponding changes in the single-ion anisotropy of the Fe­(II)­hs centers indicate. In the same experiments, no significant magnetic exchange interaction within the dinuclear complex was detected. The temperature-dependent solvation process is accompanied by a thermochromic effect as characterized by an increase of visible absorptions upon cooling leading to an observable color change from pale green to dark blue. Electrochemical studies using a LiCl/MeOH electrolyte medium to suppress speciation reveal two distinct redox processes attributed to a symmetric di-iron complex. The observation of one reversible redox process close to the ferrocene couple highlights the principal redox stability of this new di-iron system and thus its ability to mediate electron transfer reactions.^21–24^

**Figure 1:**
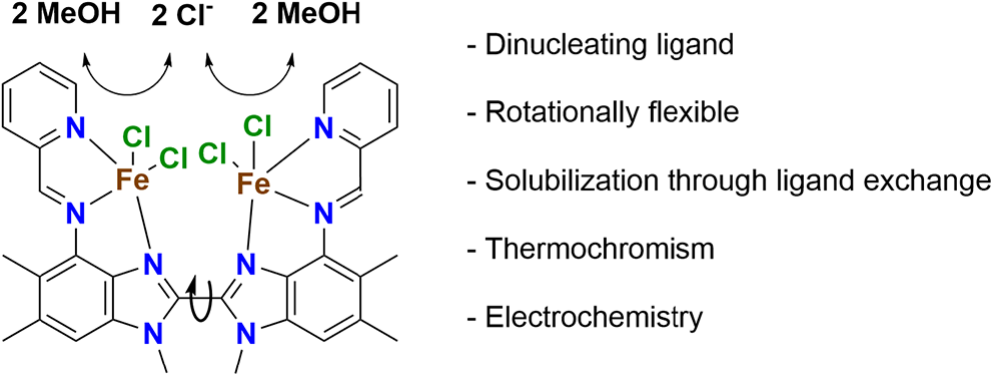
Graphical summary of the key concepts presented in this work.

## Experimental Details

All details concerning the synthetic protocols are given in the ESI.

### Computations

All geometry optimizations were performed using the meta-GGA M06L functional^25^ with a Def2-SVP basis set as implemented into Gaussian16 Revision C.01.^26^ This was followed by single-point computations using the more accurate M06/Def2-TZVP setup.^25^ M06 possess a moderate amount of exact exchange (27%) and therefore allows for optimal error cancellation between dynamic and static correlation errors.^27^ Following this work, an error of approximately 7 kcal/mol can be expected. Solvation was included through the implicit SMD model with parametrization for methanol.^28^ Solvation energies obtained using implicit solvation models are well-known to only be of moderate accuracy. This is especially problematic for charged species.^29^ We therefore corrected the computed Gibbs free energy of dissolution of Cl^–^ in methanol to reproduce the experimental value of −81 kcal/mol.^30,31^ For the sake of consistency, the experimental value of −9 kcal/mol was used for the dissolution of methanol in methanol.^32^ This procedure was used to evaluate the ligand-exchange mechanism to obtain more reliable data for the reaction with varying number of Cl^–^ and methanol ligands. Geometries were considered converged if no imaginary modes were present. Enthalpy, entropy, and zero-point-energy corrections were extracted from the geometry optimization step at the respective M06L/Def2-SVP/SMD level of theory while total energies were taken from the hybrid level computations. Activation barriers were estimated using the method of Hartwig and Hall.^33^ Temperature dependence of the Gibbs free energies was computed through eq 1

1 $$G=E+H_{corr}-T\Delta S$$

where *E* is the total electronic energy, *H*_corr_ is the enthalpy correction including the zero-point energy, and *T*Δ*S* is the entropy at temperature *T*. The temperature dependence of the enthalpy is only minor and thus neglected. A correction factor of 1.89 kcal/mol was added to convert the computed Gibbs free energies from 1 bar to the standard state of dissolved species which is 1 mol/L.^34^ Concentration dependence was included through the change in the Gibbs free energy (eq 2)­

2 $$G=G^{{}^{\circ}}+\mathrm{RT}\;\ln\left(\frac{\left[Cl^{-}\right]}{{\left[Cl^{-}\right]}^{{}^{\circ}}}\right)$$

where *R* is the general gas constant, *G* ^o^ is the Gibbs free energy under standard conditions, and [Cl^–^] is the concentration of chloride in the solution normalized by the concentration under standard conditions of 1 mol/L ([Cl^–^]°).

BS-DFT calculations were run using ORCA v6.0.1, hybrid functional M06, basis set def2-TZVP, and structure coordinates from the crystal structures of {Fe_2_Cl_4_}·THF and {Fe_2_Cl_2_}­Cl_2_ with prior optimization of H atom positions. For enigmatic reasons the calculation of the latter would only converge using an SMD solvent model, which does no significant changes to the resulting coupling constants as was checked in analog calculations with and without solvent model on {Fe_2_Cl_4_}·THF.

### Magnetometry

Magnetic measurements were performed on a Quantum Design MPMS-XL SQUID magnetometer on polycrystalline samples ground and transferred to the gelatin capsule in a flowless N_2_-filled bag and subsequently transferred quickly into the sample chamber at 2 K. Susceptibility data were obtained in the temperature range from 2 to 250 K with a static field *H*_dc_ of 0.2 T. Magnetization data were collected from 2 to 5 K up to 5 T magnetic field. The collected data were corrected for the diamagnetism of the sample holder, the capsule, and the diamagnetic contribution of the ligands. The MagProp module within the program package DAVE^35^ was used to perform full-matrix diagonalization altering iteration fittings of susceptibility and magnetization data.

### Electrochemistry

Electrochemical measurements for cyclic voltammetry were performed in a three-electrode cell consisting of a platinum wire counter electrode, a silver wire pseudoreference electrode (Ag/Ag^+^), and a glassy carbon working electrode (diameter of 3 mm). The measurements were performed with a PINEWavePico wireless potentiostat using AfterMath software. All measurements were performed inside of a Bruker glovebox operating under a nitrogen atmosphere. Typically, 10 mL of the total solution was employed, with 0.1 M concentration of the supporting electrolyte (tetrabutylammonium hexafluorophosphate (Bu_4_NPF_6_) for organic molecules and LiCl for measurements of chloride metal complexes to avoid ligand exchange) and 1 mM concentration of the analyte in the corresponding solvent. Ferrocene (Fc) was used as an internal reference unless otherwise indicated, therefore, potentials are relative to the Fc/Fc^+^ redox couple. A scan rate of 100 mV/s was employed.

## Results and Discussion

### Ligand Synthesis

The dinucleating ligand ^**Me**^ **bpbbi** was synthesized on a multigram scale over five steps starting with (1) the condensation of 4,5-dimethyl ortho-phenylenediamine with trichloroacetic acid to form the base 5,5′,6,6′-tetra-methyl-2,2′-bibenzimidazole. Subsequent oxidation of the 4,4′-positions (2) yields the dinitro derivative, followed by methylation (3) of the 1,1′-positions (imidazole amines), reduction of the nitro groups (4) to form the 4,4′-diamino derivative which finally undergoes double condensation with 2-picolincarbaldehyde to yield ^**Me**^ **bpbbi** (5). Recrystallization from toluene yielded crystals suitable for single-crystal X-ray diffractometry (scXRD), confirming the predicted composition (see Figure 2 and ESI). The bibenzimidazole is oriented such that the two tridentate 4′-picoliniminobenzimidazole ligand spheres are opposite to each other in a planar bibenzimidazole framework (Figure 2). The torsion angle as determined through the imidazole and central C–C single bond (N1–C1–C1′-N1′, N2–C1–C1′-N2′) deviates only slightly from an ideal 180° by 4° (175.1(1)°, 176.9(1)°), hence placing the two potential tridentate coordination spheres opposite to each other. Short contacts between the imino–C-H function and the benzimidazole-imine is furthermore indicative of a dipole–dipole interaction between the slightly acidic proton and the lone-pair of the imine. Notably, this interaction is different for the two imino functions: on one side, the picolinimine is rotated out of plane from the respective benzimidazole at an angle of 25.57° and C–H···N distance (between non-H atoms) is 2.923(2) Å. On the other side, the picolinimine/benzimidazole angle is much larger at 56.25°, which facilitates an intermolecular C–H···N interaction at a distance of 3.418(2) Å in addition to an intramolecular C–H···N distance of 3.026(2) Å. The intermolecular interaction connects two molecules of ^**Me**^ **bpbbi** into a π-stacked dimer with a stacking distance of 3.465 Å.

**Figure 2:**
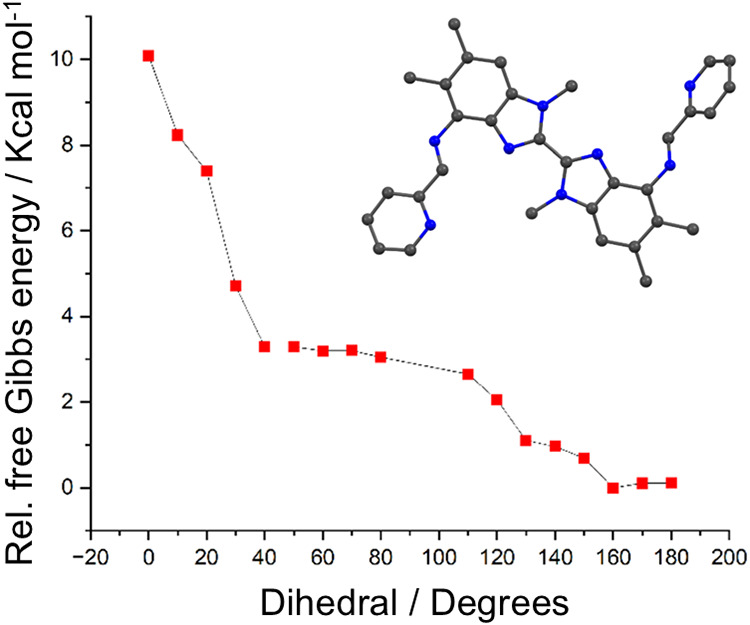
Computational scan along the potential energy surface (PES) spanned by ligand rotation. The inset shows the solid-state structure of ^**Me**^ **bpbbi** obtained through scXRD analysis. Color code: black: carbon; blue: nitrogen; hydrogen is not detectable in scXRD and therefore not shown.

To estimate the potential rotational barriers for the rotation around the central C–C single bond which could potentially inhibit the metal–metal interaction in dinuclear metal complexes, the molecular structure was also optimized by DFT. As observed in the solid state, the optimized structure also deviates slightly from planarity with a torsion angle of 160°. Intramolecular C–H···N short contacts are also observed. A potential energy surface (PES) scan was performed by optimizing the ligand geometry at fixed torsion angles for the central single C–C bond in steps of 10° (Figure 2). According to these optimizations, the optimal angle is 160°. Forcing the ligand in a completely planar structure at 180° results only in a negligible decrease of the relative stability, i.e., the change in the Gibbs free energy is close to 0 kcal/mol. Notably, this is in contrast to the solid-state structure of the respective nonalkylated 4,4′-bispicolinimino-5,5′,6,6′-tetramethyl-2,2′-bisbenzimidazole reported by Grüßing et al., in which the bisbenzimidazole is perfectly planar.^36^ This is opposed to the rotation toward 0°, i.e., a planar configuration with the picolinimine substituents on the same side, which results in a steady decrease of the stability owing to an increase of steric interactions between the methyl groups. The completely planar structure at 0° is finally the least stable configuration by 10 kcal/mol due to collision of the two methyl substituents in the 1,1′-positions. Hence, steric repulsion in the backbone keeps the two tridentate coordination spheres spatially separate and increases the likelihood for the coordination of two separate metal centers.

### Complex Synthesis

Coordination of iron­(II) chloride was performed in dry THF under a protective N_2_ atmosphere since the absence of moisture was, in accordance with the literature, found to be critical to prevent hydrolysis of the imine C = N bond of the ligand.^19^ To a suspension of the yellow ligand was added a suspension of anhydrous FeCl_2_. A gradual color change from yellow to pale green and dissolution of the iron precursor upon stirring for 16 h at room temperature indicated the formation of the anticipated complex (reaction 3).
3

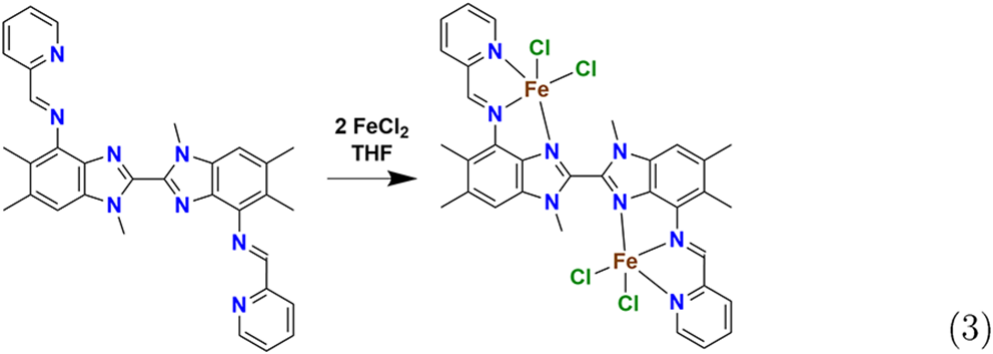




The green suspension was subsequently filtered through a glass frit, washed with THF and ether, and dried *in vacuo*. The new compound was found to be insoluble in organic solvents with low polarity, specifically diethyl ether, THF, and toluene, while attempts to dissolve the compound in polar solvents, namely, acetonitrile, DMF, or DMSO, led to decomposition as indicated by a loss of the green color and precipitation of the free ligand. Fortunately, and somewhat surprisingly, the compound was found to dissolve well in methanol without any signs of decomposition, as it forms a dark bluish-green solution at room temperature.

### Structural Characterization

Vapor diffusion of THF into the dark-green solution of the complex leads to precipitation of a dark-green crystalline solid. Slowing this process by decreasing the cross-section of the vapor diffusion path allowed for the formation of crystals suitable for scXRD analysis. The respective solid-state structure confirms the expected structure of the new di-iron complex {**Fe**_**2**_} as the THF solvate {**Fe**_**2**_}·**THF** (Figure 3a). The compound crystallizes in the monoclinic space group *P*2_1/_ *c* with four units of {**Fe**_**2**_} and four molecules of cocrystallized THF per unit. Both metal ions show a distorted pentacoordinate square-pyramidal geometry with the tridentate picoliniminobenzimidazole coordinating in a meridional fashion (Figure 3a). However, there is no crystallographic symmetry superimposing the two halves of the dinuclear complex despite their similar geometry. The respective τ_5_ values, which describe the transition between fully square-pyramidal (τ_5_ = 0) and trigonal bipyramidal (τ_5_ = 1), are 0.105 (Fe01) and 0.022 (Fe02), highlighting an unexpected asymmetry between the two complex halves which was also observed in the free ligand (see above). It is noted that the largest angle is not N_py_–Fe-N_BI_ which would be expected to be close to 180° based on the meridional coordination of the picoliniminobenzimidazole, but the N_imine_–Fe-Cl_eq_ angle which emphasizes that this geometry is best described as distorted square-pyramidal. The dihedral angle around the central C–C bond is 111.7°, putting the two iron centers at a distance of 6.570(1) Å. Hence, this shows that the two ligand spheres can be regarded as separate from each other without any sign of direct metal–metal interactions, neither direct nor through potential bridging ligands.

**Figure 3:**
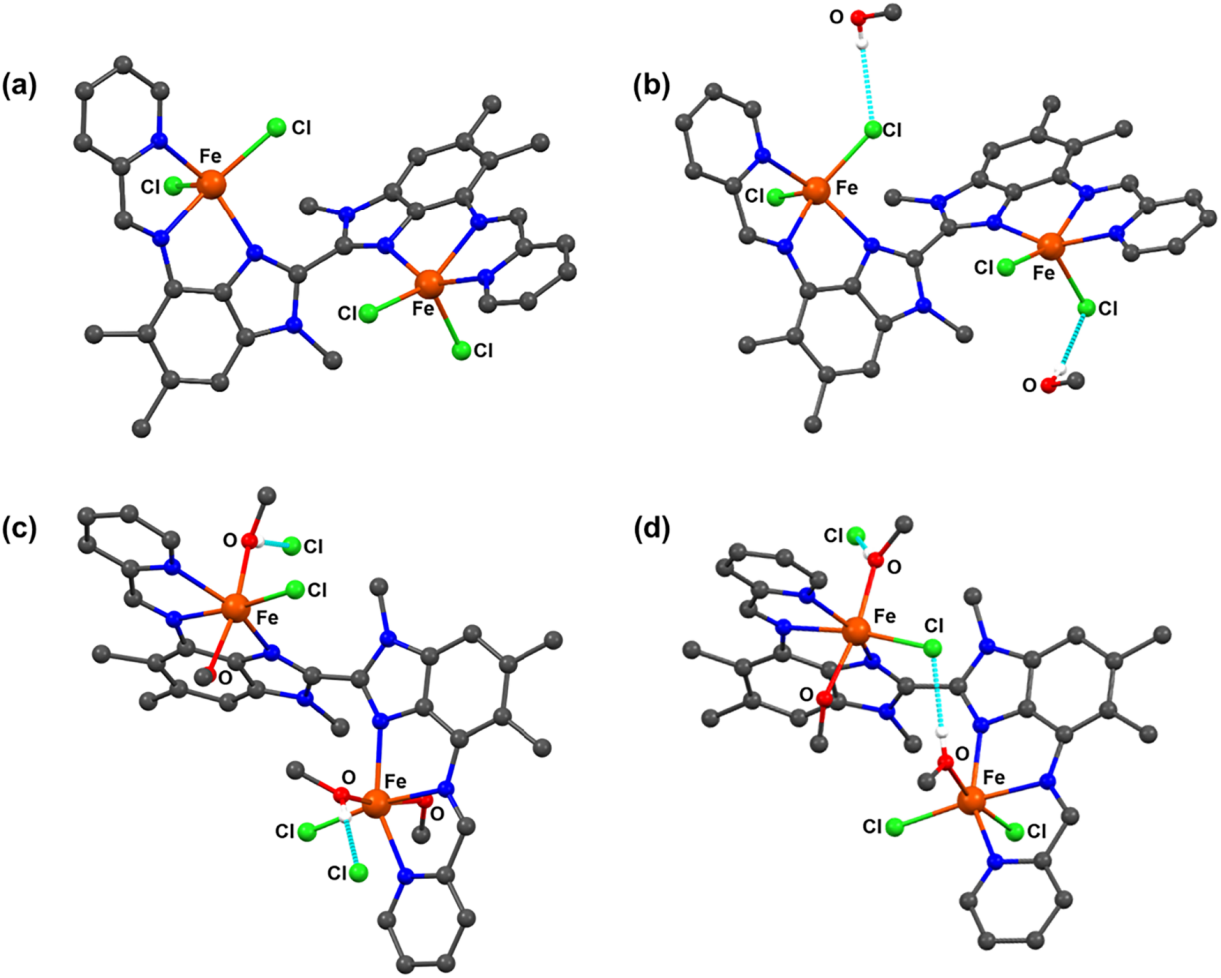
Solid-state structures obtained from methanol solutions of {**Fe**_**2**_} under conditions as follows; (a) vapor diffusion of THF into a methanol solution, one molecule of THF per complex cocrystallized, {Fe_2_Cl_4_}·THF; (b) vapor diffusion between methanol solution and toluene as nonsolvent; two methanol molecules cocrystallized and bound to coordinated chlorido ligands through hydrogen bonds as indicated, {Fe_2_Cl_2_}_2_MeOH; (c) liquid–liquid diffusion between methanol solution layered with diethyl ether, crystallization at −40 °C, {Fe_2_Cl_2_}­Cl_2_; (d) vapor diffusion of diethyl ether at −40 °C, {Fe_2_Cl_3_}­Cl; all structures shown as ball-and-stick depictions, solvent molecules and hydrogen atoms unless involved in hydrogen bonds omitted for clarity. Color code: White—H; gray—C; green—Cl; red—O; blue—N; orange—Fe.

When toluene was used as the nonsolvent instead of THF in the vapor diffusion crystallization, dark-green crystals suitable for scXRD analysis were also obtained. However, under these conditions {**Fe**_**2**_} crystallizes in the triclinic *P*1̅ space group as a methanol solvate. Co-crystallization of methanol reveals hydrogen bonding interactions with the coordinated chlorido ligands (Figure 3b). This interaction is again asymmetric as methanol binds to the planar chloride in *trans* position to the imine on Fe02 (τ_5_ = 0.12), whereas the other solvate binds to the axial chloride on Fe01 (τ_5_ = 0). This interaction also leads to a notable elongation of Fe–Cl bonds of approximately 3.6% from 2.3688(6) Å compared to 2.2856(5) Å in Fe01, and 2.3688(4) Å compared to 2.2788(4) Å in Fe02 as a consequence of the newly formed hydrogen bond between Cl and methanol. The metal–metal distance differs from that observed in the THF solvate structure at 6.8192(5) Å with a corresponding torsion angle of 126.6°, highlighting the structural flexibility of the ligand.

When attempting to crystallize {**Fe**_**2**_} at low temperature, a striking color change from pale green at room temperature to dark blue at – 40 °C was observed (see Figure 4). Contrary to intuition, this also seemed to correlate with an increase in solubility upon cooling. Unfortunately, warming the solution again from −40 °C could not be used to obtain a crystalline solid since {**Fe**_**2**_} precipitates spontaneously as an amorphous pale green solid. Crystallization was hence achieved through layering a methanol solution at −40 °C with diethyl ether. Large blue crystals suitable for scXRD analysis were obtained within 1 day. The solid-state structure reveals both metal centers to be coordinated by two molecules of methanol in addition to one chloride in *trans* position to the imine of the tridentate picoliniminobenzimidazole ligand (Figure 3c). This observation provides an obvious rationale for the increased solubility upon decreasing the temperature, namely, as a consequence of the formation of a ternary ion pair through the substitution of one chloride ligand per metal center by two solvent molecules. However, the fact that this ligand substitution reaction is favored by cooling rather than heating was rather surprising. In the solid state, two chloride counterions are bound to the complex through hydrogen bonds with the coordinated methanol. The Fe–Fe distance in this structure is rather large at 6.523(4) Å corresponding to a ligand torsion of 119.1° to accommodate the added ligands taking up space in the iron coordination spheres.

**Figure 4:**
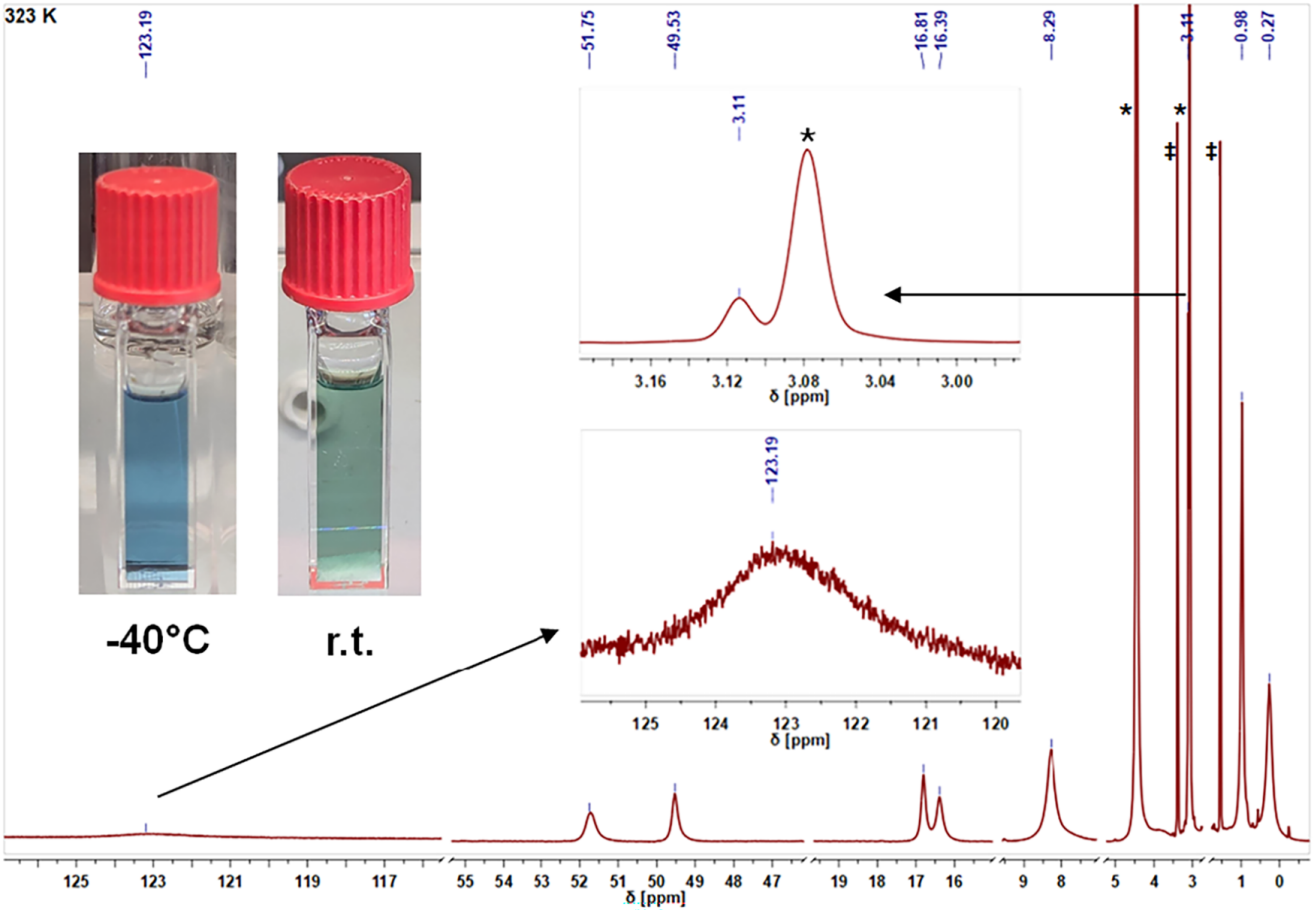
^1^H NMR spectrum recorded in methanol-d4 at 50 °C, inlays highlighting resonances at 123.19 and 3.11 ppm, solvent residual signals of methanol indicated by an asterisk (*), signals referring to cocrystallized THF indicated by the symbol ‡, the full spectrum can be found in the ESI; inlayed image showing UV/Vis cuvettes containing a methanol solution of Fe2 at −40 °C (left), and at room temperature (right).

A second polymorph of {**Fe**_**2**_} was obtained through vapor diffusion of diethyl ether into a methanol solution of {**Fe**_**2**_} at −40 °C, as opposed to liquid–liquid layering. Remarkably, this structure reveals an even less symmetric intermediate for the aforementioned ligand-exchange reaction where one iron center is already coordinated by two methanol ligands and one chlorido ligand *trans* as described before, whereas the other metal center is coordinated by only one methanol ligand, while the two chlorido ligands are still both bound to the iron center (Figure 3d). The Fe–Cl bond for the chlorido ligand *trans* to the incoming methanol is exceptionally elongated at 2.510(1)­Å, which represents a remarkable 8.3% increase compared to the Fe–Cl bond *trans* to the imine (2.317(1)­Å). Concomitantly, this weakly bound chloride ligand binds to a seemingly inbound molecule of methanol through a hydrogen bond. Direct comparison of the two low-temperature-grown structures hence seemingly reflect the “before-and-after” image of the ligand-exchange reaction (see ESI). Another notable feature of this intermediate structure is the formation of an intramolecular hydrogen bond between the methanol ligand coordinated to Fe01 and the chloride ligand on Fe02 establishing a relatively short Fe–Fe distance of 5.6968(7)­Å corresponding to a ligand torsion of 112.8°. This observation highlights how rotation around the central C–C single bond enables direct interaction and potential cooperativity between the two coordination spheres.

To further elucidate the principal ability of this ligand system to enable varying degrees of metal–metal interactions through rotation around the central C–C single bond, we performed a PES scan for {**Fe**_**2**_} in 10° steps (Figure 5). The potential energy diagram shows two distinct minima, namely, a slightly higher local minimum at 40° where the complex adopts a bridging bis-μ-chlorido conformation as well as a global minimum at 110°. This structure compares very well with the observed solid-state structure of the THF solvate (Table 1). This can be regarded as a consequence of the increase in rotational freedom around the central C–C single bond as opposed to a rigid bis­(μ-chlorido)-bridged form.

**Table 1:** Summary of Key Structural Parameter of the Various Crystallized Complexes Compared to the Converged DFT Structure

	d(Fe–Fe) [Å]	∠(BBI)	τ_5_	d(Fe–Cl) [Å]	d(C = N) [Å]	d(Fe-{N_3_}) [Å]
				2.312(1)		
{Fe_2_Cl_4_}·THF	6.570(1)	111.7°	0.105 (Fe01)	2.315(1)	1.280(6)	0.528
			0.022 (Fe02)	2.306(2)	1.272(6)	0.393
				2.308(1)		
				2.3605(4)		
{Fe_2_Cl_4_} (MeOH)_2_	6.8192(5)	126.6°	0.12 (Fe02)	2.2788(4)	1.282(2)	0.483
			≈0 (Fe01)	2.2856(5)	1.291(2)	0.316
				2.3688(6)		
LT-{Fe_2_Cl_2_} Cl_2_	6.523(4)	119.1°		2.308(2)	1.260(4)	0.001
				2.312(2)	1.273(4)	0.052
LT-{Fe_2_Cl_3_}Cl	5.6968(7)	112.8°		2.360(1)		
				2.317(1)	1.279(4)	0.081
				2.510(1)	1.279(4)	0.198
				2.32		
DFT opt. 110°	6.46	111.3°	0.128	2.32	1.3068	0.434
			0.128	2.32	1.3068	0.433
				2.32		

**Figure 5:**
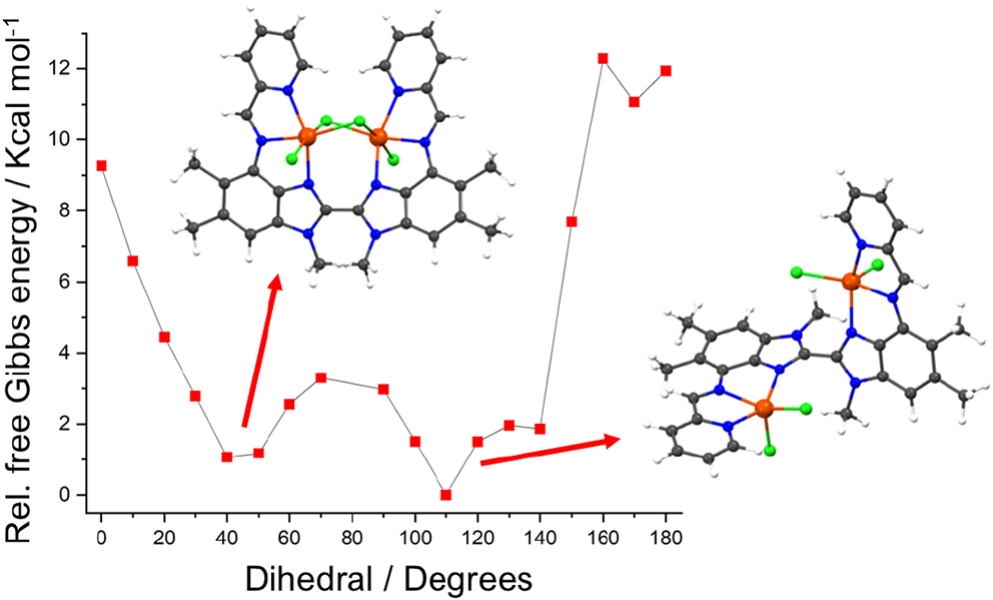
Computed PES scan along the central C–C bond rotation in steps of 10 degrees; the inlayed structures represent local minima, namely, a bridged structure at a rotation angle of 40°, and an unbridged structure which closely resembles the structures found in the solid state (see [Table tbl1]).

Comparison of enthalpy and Gibbs free energy suggests that the nonbridged configuration is slightly favored by entropy contributions. Areas of high potential energy are at 0° as observed for the ligand, and around 80° as methyl groups in the 1,1′-positions of the ligand clash with the coordinated chloride ligands. At 180°, the potential energy increases, identical to the pure ligand, to 10.0 kcal/mol relative to the global minimum. This is again due to direct steric hindrance between the metal center and the methyl substituents in the 1,1′-positions as was already demonstrated for the free ligand (see ESI). The local maximum at 70° corresponds to a minor activation barrier of 2.2 kcal/mol, which separates the bridged and nonbridged configurations. In both solid-state structures, in contrast, these methyl groups are locked in between the two chloride ligands of the opposite complex fragment.

^1^H NMR spectroscopy was employed to characterize the complex in solution. The ^1^H NMR spectrum recorded in methanol-d_4_ shows paramagnetically shifted resonances in a range from 130 to 0 ppm in accordance with the expected high-spin nature for iron­(II) in an N_3_Cl_2_ coordination sphere {**Fe**_**2**_}. Using the Evans NMR method, an effective magnetic moment μ_eff_ of 4.6 μB was determined per iron center which is lower than the spin-only value of 4.90 μB expected for an S = 2 spin state. However, at room temperature, several areas of the NMR spectrum are poorly resolved, indicating the presence of several species in solution. Considering the different ligand-substituted species observed in the solid state, we consequently conducted variable temperature (VT)-NMR spectroscopic investigations. Indeed, upon warming to 50 °C the ^1^H NMR spectrum shows a total of nine well-defined resonances. The spectrum is therefore in good agreement with a symmetric structure as is observed in the solid-state structures obtained from room temperature-grown crystals. Six resonances at 123.19, 51.75, 49.53, 16.81, 16.39, and 3.11 ppm are clearly distinguished with roughly equal integrals (Figure 4), and are hence attributed to aromatic protons from the pyridine and imine. The remaining resonances from aromatic protons are attributed to pyridine without further specification. Resonances with relative integrals of ≈3 are located at 8.29, 0.98, and 0.27 ppm and are hence attributed to the methyl groups in the 1,1′, 5,5′, and 6,6′-positions of the ligand. The poor resolution due to additional signals appearing in the range from 10 to 6 ppm as well as 3 to 1.7 ppm below 50 °C indicates speciation which is rationalized by the substitution and dissolution of chloride ligands by the solvent, i.e., methanol, as observed in the solid-state and described by our DFT investigations detailed below.

### Electronic Properties

As mentioned above, cooling solutions of {**Fe**_**2**_} is also characterized by a color change from pale green to dark blue (Figure 6), and these colors are reflected by the crystalline solids. The UV/Vis spectrum recorded at room temperature shows a weak and broad absorption in the visible-light regime, corresponding to the observed pale green color. The absorption spectrum features a maximum at λ_max_ = 651 nm with an extinction coefficient of ≈ 760 L mol^–1^ cm^–1^ as well as two shoulders centered at 570 and 600 nm (Figure 6). Based on the extinction coefficient these absorptions can be assigned to either comparably strong d-d transitions or weak MLCT transitions, assuming a charge injection from the metal into a π* orbital of the ligand, most likely the picolinimine. Since cooling is associated with a change of the coordination sphere from distorted square-pyramidal to octahedral, one might expect a decrease of d-d transitions. In contrast, an increase of the visible-light absorption intensity upon cooling is observed. This observation is hence more in line with MLCT absorptions as the metal ions align better in the plane of the mer-coordinating ligand in the octahedral structures ([Table tbl1]), hence increasing the overlap between metal-d and ligand-π orbitals. The noticeable color change when the solution is cooled to −40 °C is characterized by an increase of the absorption maximum at 651 nm along with a concomitant increase of the absorption intensities in the range between 500–600 nm accounting for the perceived green-to-blue color change. This was further tracked through recording of a series of UV/vis spectra during warming from −40 °C to room temperature which revealed two distinct isosbestic points at 280 and 495 nm, suggesting the transition between two distinct electronic structures (Figure 6 and Figure S23). In order to conclusively correlate the UV–Vis data with the clean high-temperature structure observed by NMR, further UV–Vis measurements were conducted between 20 and 50 °C, showing only a marginal decrease of the visible absorption band. To better understand the full picture, DFT modeling was employed to calculate the relative stability of different possible species based on the structures which were observed in the solid-state. The observed temperature dependence leading to ligand substitution and a concomitant increase in solubility suggest that the underlying process is entropy-driven.

**Figure 6:**
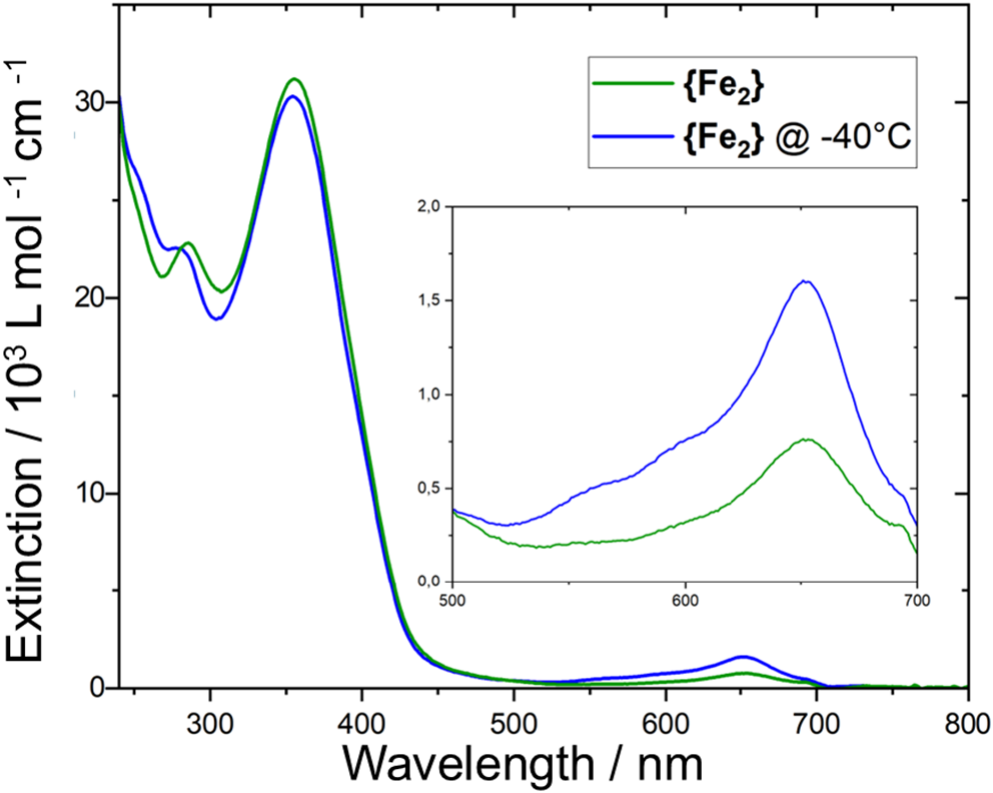
Changes to the UV/Vis spectra upon cooling from room temperature to −40 °C.

Based on XRD results (Figure 3) it is reasonable to assume the exchange of two chlorides by four methanol ligands. In line with experiment, we find that the ligand exchange of 2 Cl^–^ by 4 methanol is strongly favorable at very low chloride concentrations while it becomes thermodynamically unfavorable at very high concentrations both at high and low temperatures which effectively inhibits this reaction. At intermediate concentrations finally this reaction is only favored at lower temperatures (see ESI for comparison of the driving forces at varying Cl^–^ concentrations). According to our computations, the differences in stability are purely entropy-driven. Note that the underlying computations and models are subject to computational uncertainties which will affect the exact crossover points to conditions where ligand exchange is favored. Furthermore, differences in activation barriers and binding energies are often of the order of the expected error bars. Thus, the shown mechanism corresponds to a qualitative reaction sequence. In the absence of an experimentally determined Cl^–^ concentration, we opted to illustrate relevant trends using a concentration of 10^–9^ mol/L when evaluating the detailed mechanism of ligand exchange.

A summary of the most likely mechanism and the path energetics is shown in Figures 7 and 8.

**Figure 7:**
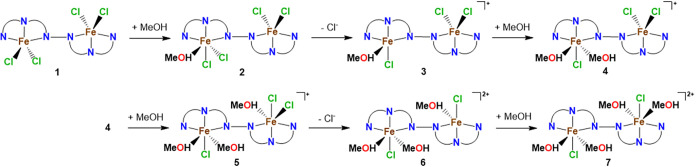
Summary of the anticipated qualitative ligand-exchange sequence based on the scXRD measurements and DFT computations.

**Figure 8:**
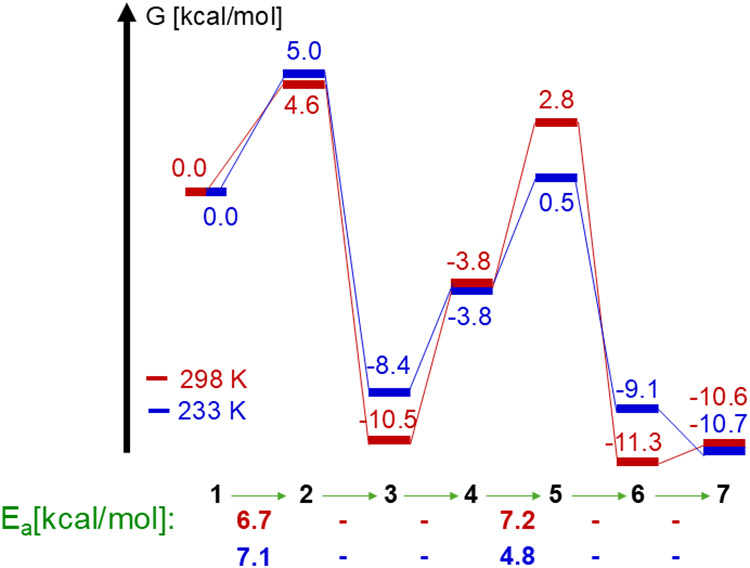
Summary of the computed binding energies of all anticipated intermediates. Since the exact Cl^–^ concentration could not be determined experimentally, a Cl^–^ concentration of 10^–9^ mol/L was assumed. Activation barriers were estimated using the method of Hartwig and Hall.^33^ A semiquantitative accuracy is expected. A ”–” indicates that the process is barrierless, i.e., the estimated barrier was lower than the reaction energy.

The reaction is initialized by the coordination of a methanol molecule to one of the Fe centers. This reaction is, owing to the significant reduction of local entropy, slightly uphill by roughly 5 kcal/mol with a barrier of 7 kcal/mol at either temperature. The subsequent release of Cl^–^ is then moderately exergonic by approximately −9 kcal/mol with a spurious barrier of 1 kcal/mol. Naturally, this step is strongly dependent on the chloride concentration, i.e., it becomes less favorable with higher chloride concentrations. According to our computations, the Fe-dimer rotates slightly to form a very strong intramolecular H-bond between the methanol OH group and Fe–Cl (d­(OH···ClFe)=2.26 Å). This slightly weakens the Fe–Cl bond as indicated by an increase of the Fe–Cl bond length by 0.05 Å. This is followed by the coordination of a second methanol to the same Fe center. This step is again somewhat endergonic by 7 kcal (298 K) and 5 kcal/mol (233 K) with a total barrier of 10 kcal/mol at room temperature. The same reaction sequence of methanol coordination, chloride release and binding of a second methanol is then repeated at the second metal center. Overall, the energetics are qualitatively similar albeit all steps are thermodynamically slightly less favorable (Figure 8). It is noteworthy, that the scXRD measurements suggest a very strong hydrogen bond between methanol and Cl^–^ for intermediate **5** which could aid the Cl^–^ release. However, we were unable to reproduce this structure in our computations, i.e., they always yielded a structure displaying a strong MeOH···MeOH hydrogen bond even when using the scXRD structure as input. Closer evaluation of the geometry optimization path suggests that the scXRD structure is energetically equivalent to a MeOH···MeOH H-bond with no major barriers in between, i.e., it corresponds to a shallow minimum at the potential energy surface. Note that all attempts to stabilize this structure without the presence of minor imaginary modes failed due to the facile energy differences between either configuration. This deviation is indeed not completely surprising when considering the very different embedding of the complex in the crystal structure and in solution. According to our computations this reaction sequence is subject to very low barriers for the initial steps and moderately high barriers for the final 2 steps. Additionally, all steps are either thermodynamically favorable or in very few cases only slightly endergonic both at 233 and 298 K. Further support for the mechanism derived from DFT modeling comes from the scXRD measurements which confirm the existence of the intermediates **1**, **5** and **7** (Figure 3). Following this mechanism, an increase in chloride concentration should easily push the observed equilibrium toward the more symmetric structure.

### Magnetometry

Given the high-spin nature of the complexes found in the NMR spectra, we performed temperature-dependent magnetic susceptibility as well as magnetization measurements. We first explored the magnetic conduct of polycrystalline {Fe_2_Cl_4_}·THF to investigate potential magnetic interactions within the compound. The measured molar magnetic susceptibility (Figure 9) of the complex at 250 K (χ*T*_mol_ = 6.73 cm^3^Kmol^–1^) is in agreement with the expected values for two noninteracting Fe­(II)*hs* ions (2*S* = 2, χ*T*_mol_ = 6.00 cm^3^Kmol^–1^) and thereby indicating minor orbital contributions. The magnetic susceptibility stays constant until temperatures around 75 K, and at temperatures below 50 K drops abruptly. The lowest magnetic susceptibility value for this complex was measured at 2 K below χ_*M*_T = 3.00 cm^3^Kmol^–1^. The respective magnetization curves show no characteristic deviation from Brillouin functions and are depicted in Figure S34.

**Figure 9:**
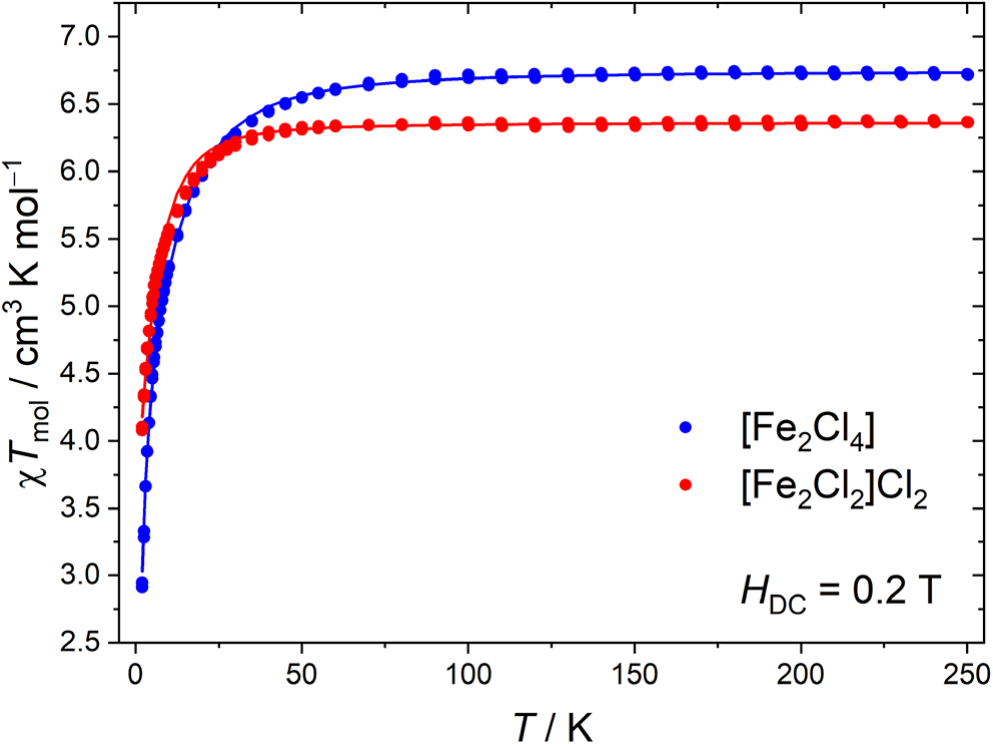
Temperature dependence of the molar magnetic susceptibility (χ*T*_mol_) for {Fe_2_Cl_4_}·THF (blue dots) and {Fe_2_Cl_2_}­Cl_2_ (red dots). The underlying lines are obtained by the fit to the model specified in the ESI.

From the parameter weighted fitting of both magnetic susceptibility and magnetization to an appropriate spin Hamiltonian (eq SI1, see ESI for further details), which models two symmetric Fe­(II)*hs* centers with one equal isotropic *g*-value, respectively, one equal set of magnetic anisotropy parameters *D* and *E* (equal for both centers) as well as an intramolecular magnetic coupling constant *J* can be extracted with adequate precision. Those simplifications are necessary, since we do not gain any orientational information from the polycrystalline bulk materials under investigation, hence further partitioning of the tensorial parameters would mean an overparametrisation of the data. Yet, the simplifications are acceptable given the structural similarity of both Fe­(II) centers. The decrease in χ_*M*_T can be attributed to moderate single-ion magnetic anisotropy as well as weak antiferromagnetic coupling (*D* = −10.09 cm^–1^, *E* = −3.35 cm^–1^, *J* = −0.05 cm^–1^). The former features a large rhombicity approaching the upper physical limit of *E*/*D* = 0.33, which is in accordance with the low-symmetry distorted square-pyramidal coordination environment of both Fe centers in {Fe_2_Cl_4_}·THF (*vide supra*). All fitting parameters are collated in Table S11.

The magnetic properties of the complex {Fe_2_Cl_2_}­Cl_2_ crystallized at low temperature were investigated under the same conditions for comparison (see Figure S35). {Fe_2_Cl_2_}­Cl_2_ follows the same general profile, although the minimum value for the for the magnetic susceptibility for this complex measured at 2 K approximates χ*T*_mol_ = 4.10 cm^3^Kmol^–1^. The parameters extracted from the experimental values fitted to the same spin Hamiltonian denote a slightly weaker antiferromagnetic coupling (*J* = −0.03 cm^–1^) and a reduced single-ion anisotropy (*D* = −5.49 cm^–1^, *E* = −0.06 cm^–1^) in comparison to {Fe_2_Cl_4_}·THF. Notably, the lower *D* as well as the vanishing *E* is in agreement with the change in coordination geometry from distorted square-pyramidal to mildly distorted octahedral shown by the scXRD data. Namely, a higher overall symmetry of the coordination environment explains the negligible *E*, while the more homogeneous ligand field also leads to roughly halfening of *D* in comparison to {Fe_2_Cl_4_}·THF.

Unfortunately, in contrast to Co­(II),^37^ literature is lacking broad structure property correlations spanning various coordination geometries for Fe­(II) or more general d^6^ ions. Merely, this is done on purely mathematic description of zero-field splitting^38^ or very basic structural models.^39^ Hence, one would have to profoundly investigate the magnetic anisotropy with theoretical tools on different structural models to further rationalize the detected trend, which is beyond the scope of this work. Therefore, the discussion has to remain qualitative here. Although the found magnetic exchange interactions *J* might appear like arbitrary fitting artifacts given their magnitude < −0.1 cm^–1^ in both cases, we can further confirm their plausibility and intramolecular origin by means of BS-DFT results generated for both crystal structures (H atoms optimized) using hybrid functional M06; basis set def2-TZVP and Yamaguchi’s approach^40,41^ adapted to the spin Hamiltonian also used for the fits (eq SI1). The results (−0.2 and −0.06 cm^–1^ for {Fe_2_Cl_4_}·THF and {Fe_2_Cl_2_}­Cl_2_, respectively) well reproduce the sign, trend and magnitude of the experimentally determined coupling constants. Moreover, these findings are in line with the neglectable electronic interaction between both iron centers as hinted by the calculational and UV–vis results.

### Electrochemistry

To gain insights into the electrochemical behavior of the complex and its potential use as an electrocatalyst, cyclic voltammetry (*CV*) measurements were performed. As a first step, we decided to study the free ^**Me**^ **bpbbi** ligand. Given the sensitivity toward hydrolysis of the imine bonds, *CV*s were recorded in dry DMF under a dry N_2_ atmosphere in the glovebox. Due to poor solubility, a supersaturated solution was prepared by careful heating to 60 °C and subsequent cooling to room temperature. The *CV* of the free ligand shows irreversible processes below −2.0 V and above 0.5 V versus Fc/Fc^+^, see ESI. The irreversible oxidation processes above 0.5 V are most likely linked to decomposition, potentially corresponding to side-reactions of a highly reactive radical cationic species. Two irreversible reductions take place at −2.32 and −2.72 V vs Fc/Fc^+^. The first peak is preliminarily assigned to the reduction of the R–C = N–R′ imine bond to form the radical anion ^**Me**^ **bpbbi** ^·–^ which would be subject to some very fast follow-up reactions precluding reversibility. To support this hypothesis, dry methanol was added as proton donor to enable the proton-coupled 2-electron reduction of the imine to form the respective amine. Indeed, the first reduction was found to shift by roughly 70 mV to a less negative potential of −2.25 V vs Fc/Fc^+^, see ESI. This is further supported by the result of reactivity studies with the hydride reagent NaHBEt_3_ which consistently yielded the respective picoliniminobibenzimidazole derivative, see ESI.

Electrochemical data of the di-iron complex {**Fe**_**2**_} were collected in dry methanol solution in accordance with its low solubility and instability in other solvents including DMF. However, using NBu_4_PF_6_ as the electrolyte led to the immediate precipitation of an insoluble blue solid. Taking into account the ligand-exchange equilibrium and the correlated thermochromic effect in methanol, the blue color of the precipitate is assigned to a hexafluorophosphate salt derivative of {**Fe**_**2**_}, which appears to be insoluble in methanol. Details on the nature of these salt derivatives are the subject of further investigation and will be described in a follow-up study. On the basis of our understanding of the ligand-exchange mechanism as described above, we opted for LiCl as the conducting salt to obtain clean electrochemical data that can be attributed to {**Fe**_**2**_}. Indeed, using a 1 mM solution of LiCl as the electrolyte in methanol yielded very clean redox processes within the accessible potential window, namely, one irreversible process at −1.38 V vs Fc/Fc^+^, as well as one reversible redox process at −0.05 vs Fc/Fc^+^ (Figure 10). A more detailed analysis of the electrochemical properties based on cyclic voltammetry and in-depth DFT evaluation of the underlying electrochemistry suggests that the reversible peak corresponds to a one-electron process.^42^

**Figure 10:**
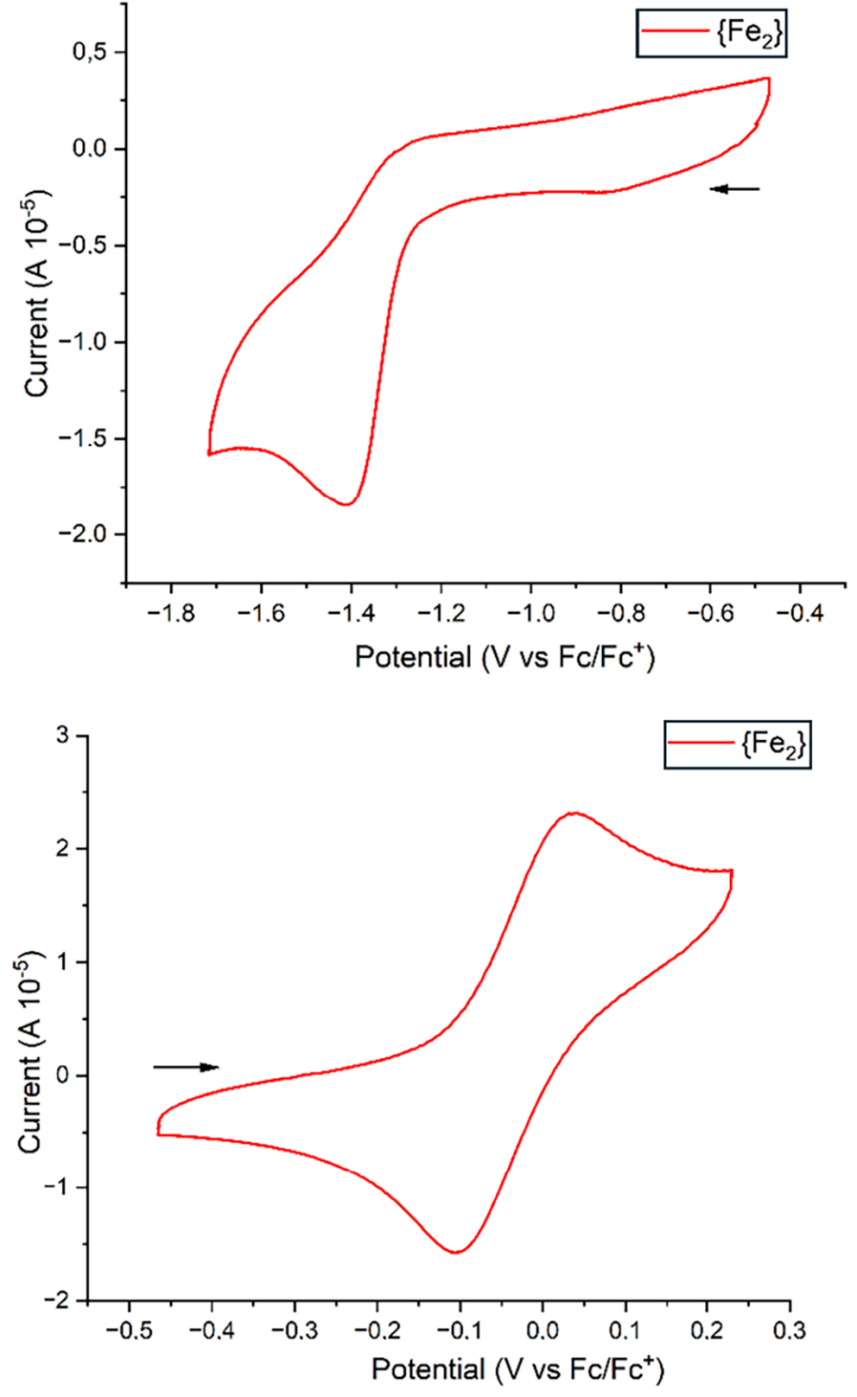
*CV* in 2 different potential ranges. All measurements were done with 1 mM of {**Fe**_**2**_} in 0.1 M LiCl dissolved in MeOH at 100 mV/s.

Following the analysis of the pure ligand, we associate the irreversible redox process with a predominantly ligand-centered reduction located on the imine bond, as described above. This process is shifted to less negative potentials compared to the ligand by 877 mV, which is in agreement with the influence of the coordinated metal ion acting as an electron-withdrawing Lewis acid. In addition, this value compares well to that of iron complexes of the redox noninnocent pyridine di-imine ligand which is typically reported at around −1.20 V vs Fc/Fc^+^.^43^ Regarding the reversible redox process found at −0.05 V vs Fc/Fc^+^, it is tempting to assign it to a purely metal-centered process which is, however, not fully supported by the available data, and, *in lieu* of appropriate experimental data, we refrain from a definitive assignment of this redox process. Within the context of this study, an understanding of ligand-exchange processes was critical to optimizing experimental conditions in order to record well-defined redox processes. A more detailed study focusing on the electrochemistry of ^**Me**^ **bpbbi**-supported midiron complexes in different media is currently being prepared.

## Conclusions

We report the synthesis and characterization of a new di-iron complex, namely {Fe_2_}, based on the rotationally flexible ^**Me**^ **bpbbi** ligand framework. Through a combination of studies in solution, *in crystallo*, and *in silico*, we describe an unexpected entropy-driven ligand-exchange process in methanol solution. This speciation leads to a counterintuitive increase in solubility upon cooling which is accompanied by a thermochromic effect due to the underlying changes in the complexes ligand spheres. Importantly, structural changes are supported by a rotational rearrangement of the supporting ligand, highlighting a unique feature of this dinucleating ligand. Intermediates of the mechanism were identified based on solid-state structures obtained from crystals grown at different temperatures. These solid-state structures were also harmonized with magnetometry data revealing their magnetic anisotropy in dependence of the coordination environment and verifying negligible magnetic exchange between the two Fe­(II) centers. By means of DFT modeling a reaction mechanism was constructed. Computations and temperature dependence suggest that the exchange mechanism is driven by minor decrease in entropy due to replacing 2 chlorides by 4 methanol molecules. Electrochemical characterization under optimized conditions reveals two well-defined redox processes, including a reversible process at −0.005 V vs Fc/Fc^+^, might show promise for future applications in redox catalysis.^21–24^ By integrating structural, computational, magnetometric, and electrochemical analyses, this work provides a comprehensive understanding of ligand-exchange dynamics in dinuclear iron complexes, offering new insights into their design for catalytic applications inspired by biological systems.

## Supplementary Material



## Notes

Zenodo archive: raw data of DFT computations including final structures, Gibbs free energies and electronic energies (DOI: 10.5281/zenodo.16892965; textfile/ZIP archive); supporting information: synthetic protocols, chemical and electrochemical characterization of ligand and complex, analysis of influence of Cl^–^ concentration on calculated reaction energies, alternative ligand-exchange paths, further details on magnetometric measurements (PDF); crystallographic data is available at the Cambridge Structural Database (CSD) under the deposition numbers: 2480870 (^**Me**^ **bpbbi**), 2480871 ({Fe_2_Cl_4_}·THF), 2480872 ({Fe_2_Cl_4_}·2MeOH), 2480873 ({Fe_2_Cl_2_}­Cl_2_), 2480884 ({Fe_2_Cl_3_}­Cl), and 2526015 (^**Me**^ **bpbbi**–**H**_**4**_) (PDF)
