# Supplementary material for: Entropy-Driven Ligand Exchange in a Rotationally Flexible Dinuclear Fe(II)–Fe(II) Complex
Source: Inorg Chem. 2026 Jun 17;65(26):14449–59. doi: 10.1021/acs.inorgchem.5c05481 (PMC13343462; doi:10.1021/acs.inorgchem.5c05481)
Supplement: Supplementary file 1 [file ic5c05481_si_001.pdf]

Supporting Information: Entropy-driven ligand exchange in a rotationally flexible dinuclear Fe(II)-Fe(II) complex

Pablo G. Porta,[†] Benjamin Kintzel,[‡] Birgit Weber,[‡] Michael Busch,[*,¶,§] and Dieter Sorsche[*,†]

†*Institute of Inorganic Chemistry I, Ulm University, Albert-Einstein-Allee 11, 89081 Ulm, Germany.*

‡*Institut für Anorganische und Analytische Chemie, Friedrich-Schiller-Universität Jena, Humboldtstraße 8, 07743 Jena, Germany*

¶*Department of Engineering Sciences and Mathematics, Applied Physics, Luleå University of Technology, 971 87 Luleå, Sweden*

§*Wallenberg Initiative Materials Science for Sustainability (WISE), Luleå University of Technology, 971 87 Luleå, Sweden*

E-mail: michael.busch@ltu.se; dieter.sorsche@gmx.de

Materials and methods

If not otherwise stated, all solvents and reagents were purchased from commercial providers in 95% purity or greater and used as received. Dry solvents used for inert synthesis and spectroscopy were purchased from ACROS Organics in 99.9%+ purity, transferred into a N_2-flushed glovebox and used without further purification. Ligand synthesis was carried out under air, complex synthesis and all subsequent manipulations were carried out in an MBraun glovebox workstation equipped with a -40°C freezer and flushed with N_2 in 5.0 quality purchased from mti and dried through a drierite^{T}M column.

Optical spectroscopy

UV-vis spectroscopy was carried out using a Horiba Duetta with EzSpec device placed in a N_2-flushed glovebox. Optical quartz glass cuvettes with a pathlength of 10 mm were used. An Avantes spectroscopy bundle consisting of an AvaLight light source, fibre optics, sample cell holder and an AvaSpec detector were used.

NMR spectroscopy

NMR spectroscopy was performed either on a Bruker Avance 600 MHz or Bruker Avance 400 MHz spectrometer. The shift values are given in ppm and are referenced to the corresponding solvent residual peaks. For Evans method measurements, a glass capillary containing a TMS2O solution in methanol-d_4 was added to a solution of the complex in the same matrix.

Single crystal X-ray Crystallography (scXRD)

Crystals suitable for X-ray crystallography were mounted using a MicroLoop and Perfluoropolyalkyl ether (viscosity 1800 cSt). X-ray diffraction intensity data were measured at 150 K on a Bruker D8 Quest single crystal diffractometer with a PHOTON II detector using Mo - Kα radiation (wavelength $\lambda = 0.71073$ Å). Structure solution and refinement was car-

ried out using the SHELXL package via Olex2.[S1,S2] Corrections for incident and diffracted beam absorption effects were applied using multi-scan refinements. Structures were solved by direct methods and refined against F2 by the full-53 matrix least-squares technique. The hydrogen atoms were included at calculated positions with fixed thermal parameters. All non-hydrogen atoms were refined anisotropically unless otherwise mentioned. MERCURY was used for structural representations.[S3]

Ligand Exchange Mechanisms

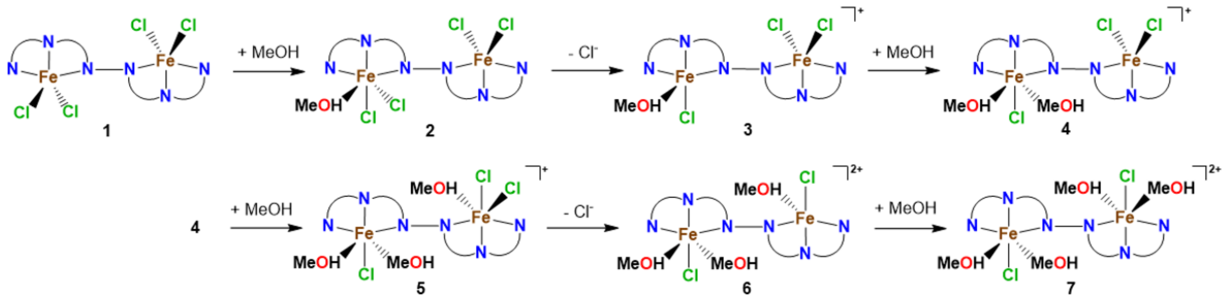

Figure S1: Most likely mechanism for ligand release based on the scXRD measurements and DFT computations.

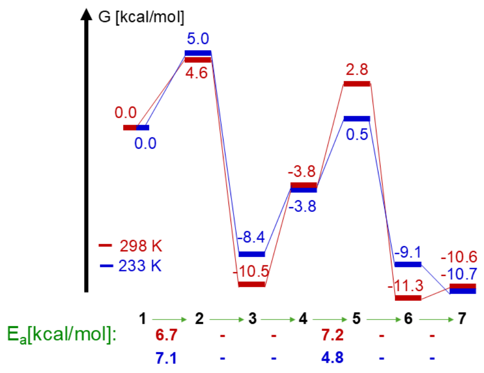

Figure S2: Computed binding energies of all anticipated intermediates for the most likely ligand exchange mechanism. The computations assume a Cl^- concentration of 10^{-9} mol/L.

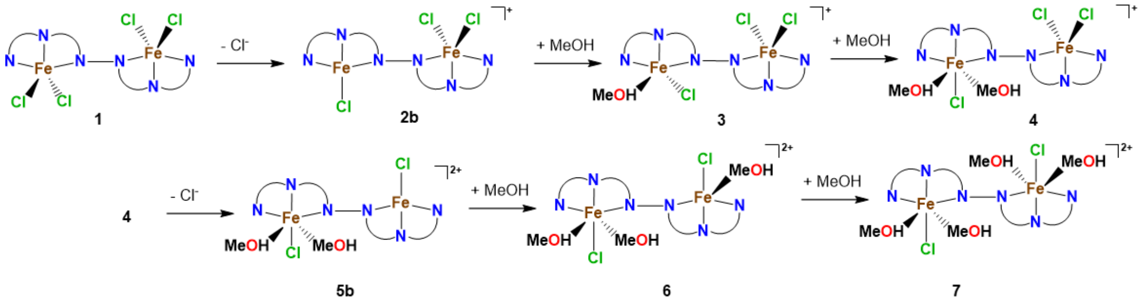

Figure S3: Alternative mechanism for ligand release based on the scXRD measurements and DFT computations.

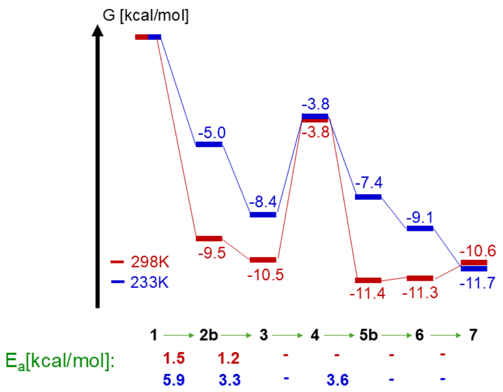

Figure S4: Computed binding energies of all anticipated intermediates for the alternative ligand exchange mechanism. The computations assume a Cl^- concentration of 10^{-9} mol/L.

$[Cl^-]$ Dependence of Ligand Exchange Mechanism

Table S1: Comparison of estimated binding energies of intermediates of the most likely reaction path at different chloride concentrations assuming a temperature of 233 K and 298 K.

	**1**	**2**	**3**	**4**	**5**	**6**	**7**
$[Cl^-] = 10^{-9}$ M							
ΔG(298K) [kcal/mol]	0	4.6	-10.5	-3.8	2.9	-11.3	-10.6
ΔG(233K) [kcal/mol]	0	5.0	-8.4	-3.8	0.5	-9.1	-10.7
$[Cl^-] = 10^{-7}$ M							
ΔG(298K) [kcal/mol]	0	4.6	-7.8	-1.1	5.6	-5.8	-5.2
ΔG(233K) [kcal/mol]	0	5.0	-6.3	-1.7	2.6	-4.8	-6.4
$[Cl^-] = 10^{-5}$ M							
ΔG(298K) [kcal/mol]	0	4.6	-5.1	1.6	8.4	-0.4	0.3
ΔG(233K) [kcal/mol]	0	5.0	-4.2	0.4	4.8	-0.6	-2.2

Table S2: Comparison of estimated binding energies of intermediates of the alternative reaction path at different chloride concentrations assuming a temperature of 233 K and 298 K.

	**1**	**2**	**3**	**4**	**5**	**6**	**7**
$[Cl^-] = 10^{-9}$ M							
ΔG(298K) [kcal/mol]	0	-9.5	-10.5	-3.8	-11.5	-11.3	-10.6
ΔG(233K) [kcal/mol]	0	-5.1	-8.4	-3.8	-7.4	-9.1	-10.7
$[Cl^-] = 10^{-7}$ M							
ΔG(298K) [kcal/mol]	0	-6.8	-7.8	-1.1	-6.0	-5.8	-5.2
ΔG(233K) [kcal/mol]	0	-2.9	-6.3	-1.7	-3.2	-4.8	-6.4
$[Cl^-] = 10^{-5}$ M							
ΔG(298K) [kcal/mol]	0	-4.0	-5.1	1.6	-0.6	-0.4	0.3
ΔG(233K) [kcal/mol]	0	-0.8	-4.2	0.4	1.1	-0.6	-2.2

PES Scan - Ligand

Table S3: Potential energy surface scan along the rotation mode of the pure ligand. Structures which displayed problematic geometry convergence behavior are marked in red.

Torsion [°]	G [Ha]	G-G_{min} [Ha]	G-G_{min} [kcal/mol]	Imaginary modes
0	-1674.3990	0.016009	10.0857	0
10	-1674.4019	0.013067	8.2322	0
20	-1674.4032	0.011737	7.3943	0
30	-1674.4075	0.007468	4.7048	0
40	-1674.4097	0.005227	3.2930	0
50	-1674.4097	0.005227	3.2930	0
60	-1674.4099	0.005070	3.1941	0
70	-1674.4099	0.005084	3.2029	0
80	-	-	-	-
90	-	-	-	-
100	-1674.3177	0.097265	61.2769	0
110	-1674.4108	0.004211	2.6529	1
120	-1674.4117	0.003257	2.0519	0
130	-1674.4132	0.001756	1.1063	0
140	-1674.4134	0.001529	0.9633	0
150	-1674.4139	0.001095	0.6898	0
160	-1674.4150	0.000000	0.0000	0
170	-1674.4148	0.000165	0.1039	0
180	-1674.4148	0.000170	0.1071	0

PES Scan - Complex

Table S4: Calculated PES along the rotation mode around the central C−C bond for $[FeCl_2]_2L$.

Torsion [°]	G [Ha]	G-G_{min} [Ha]	G-G_{min} [kcal/mol]	d(Fe-Fe) [Å]	Imaginary modes
0	-6042.945889	0.014708	9.2660	3.3170	0
10	-6042.950138	0.010459	6.5892	3.3809	0
20	-6042.953536	0.007061	4.4484	3.4413	0
30	-6042.956185	0.004412	2.7796	3.4892	0
40	-6042.958897	0.001700	1.0710	3.5348	0
50	-6042.958724	0.001873	1.1800	3.5723	0
60	-6042.956543	0.004054	2.5540	3.6024	0
70	-6042.955362	0.005235	3.2981	5.4072	0
80	-	-	-	-	-
90	-6042.955885	0.004712	2.9686	6.2313	0
100	-6042.958197	0.002400	1.5120	6.3734	0
110	-6042.960597	0.000000	0.0000	6.4630	0
120	-6042.958206	0.002391	1.5063	6.5117	0
130	-6042.957482	0.003115	1.9625	6.5279	0
140	-6042.957639	0.002958	1.8635	6.5572	0
150	-6042.948405	0.012192	7.6810	8.1950	0
160	-6042.941108	0.019489	12.2781	9.4349	0
170	-6042.943032	0.017565	11.0660	9.6977	0
180	-6042.941654	0.018943	11.9341	9.2752	0

Frontier Orbitals

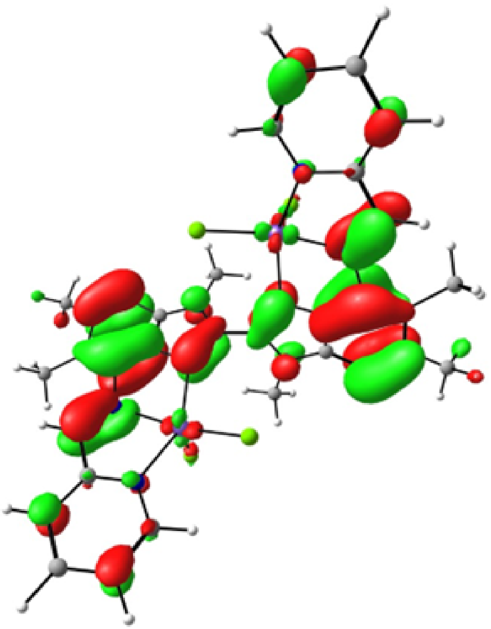

Figure S5: HOMO of {$\mathbf{Fe_2}$}. Note the bonding orbitals along the C=N bond of the imine unit.

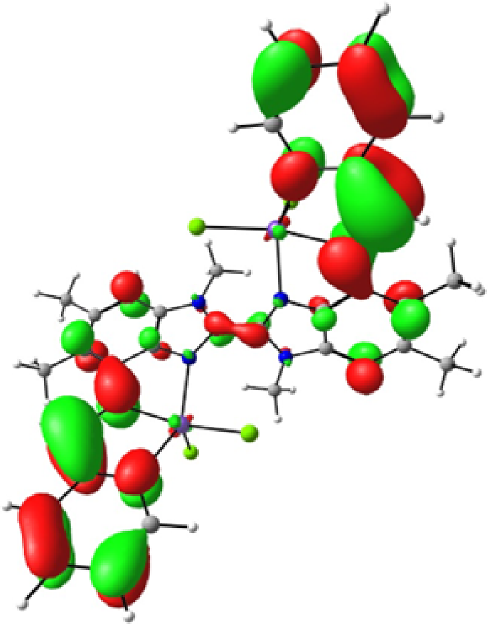

Figure S6: LUMO of {$\mathbf{Fe_2}$}. Note the antibonding orbitals along the C=N bond of the imine unit.

Computations on the spin state of the $[FeCl_2]_2L$ complex

Table S5: Calculated Gibbs energies for the possible spin states (Spin Ladder) in several oxidation states of $[FeCl_2]_2L$. Optimizations and frequency analysis were performed in gas phase using the functional M06L and def2-SVP as basis set.

Spin	G [Ha]	E [Ha]	G-G$_{min}$ [kcal/mol]	Imaginary modes
**Fe(II)Fe(II)**				
9	-6041.386507	-6041.878468	0	0
7	-6041.352324	-6041.845806	21.393848	0
5	-6041.317582	-6041.813427	43.137552	0
3	-6041.362478	-6041.855198	15.038843	0
1	-6041.272656	-6041.773077	71.255037	0
**[Fe(I)Fe(II)]$^{-1}$**				
8	-6041.496685	-6041,986505	0	0
6	-	-	-	-
4	-	-	-	-
2	-6041.494786	-6041.986307	1.191630	0
**[Fe(I)Fe(I)]$^{-2}$**				
7	-6041.578902	-6042.065489	0	0
5	-6041.549574	-6042.03842	18.403602	0
3	-6041.518171	-6042.010691	38.109287	-
1	-6041.465848	-6041.960604	70.942474	0
**[Fe(0)Fe(I)]$^{-3}$**				
6	-6041.623302	-6042.105605	0	0
4	-6041,589568	-6042.078101	21.215699	0
2	-6041,644617	-	13.375110	-
**[Fe(0)Fe(I)]$^{-3}$**				
5	-6041.608752	-6042,109600	3.660244	0
3	-6041.662393	-6042,148699	0	0
1	-6041.564365	-6042,057146	61.513514	-

Synthesis

Ligand synthesis

The dinucleating ligand **^{Me}bpbbi** was synthesized following the produre reported by Muller, Bernardinelli, and Reedijk.[S4] We note that after recrystallization of the nitration product 4,4'-dinitro-5,5',6,6'-tetramethyl-2,2'-bibenzimidazole from 2-methoxy ethanol it is imperative that the recrystallized material is thoroughly washed with methanol, followed by diethylether, and dried under vacuum to ensure removal of any protic residulas which interfere with the subsequent methylation step. Therefore, it is important to wash thoroughly the recrystallized nitrated precursor, first with methanol and thereafter with diethyl ether to remove any remaining protic solvents. After the five steps in the route, the ligand is obtained with an overall yield of 36.9%.

^{1}H-NMR (CD_2Cl_2, ppm): 10.05 (s, 1 H, imine C-H), 9.56 (s, 1 H, imine C-H), 8.79 (m, 1 H, py), 8.71 (m, 1 H, py), 8.40 (d, 1H, py), 8.00-7-70 (m, 3H, py + DMF), 7.53 (m, 2H, py), 7.38 (m, 1H, py), 7.19 (m, 1H, benz), 6.69 (m, 1H, benz), 4.32 (m, 3H, N-methyl), 4.22 (m, 3H, N-methyl), 2.51 (s, 6H, benz-methyl), 2.43 (s, 3H, benz-methyl), 2.19 (s, 3H, benz-methyl). Elemental analysis. Calculated for $C_32H_30N_8$: C, 72.98%; H, 5.74%; N, 21.28%. Found: C, 71.72 ± 0.12%; H, 5.86 ± 0.20%; N, 19.81 ± 0.10%.

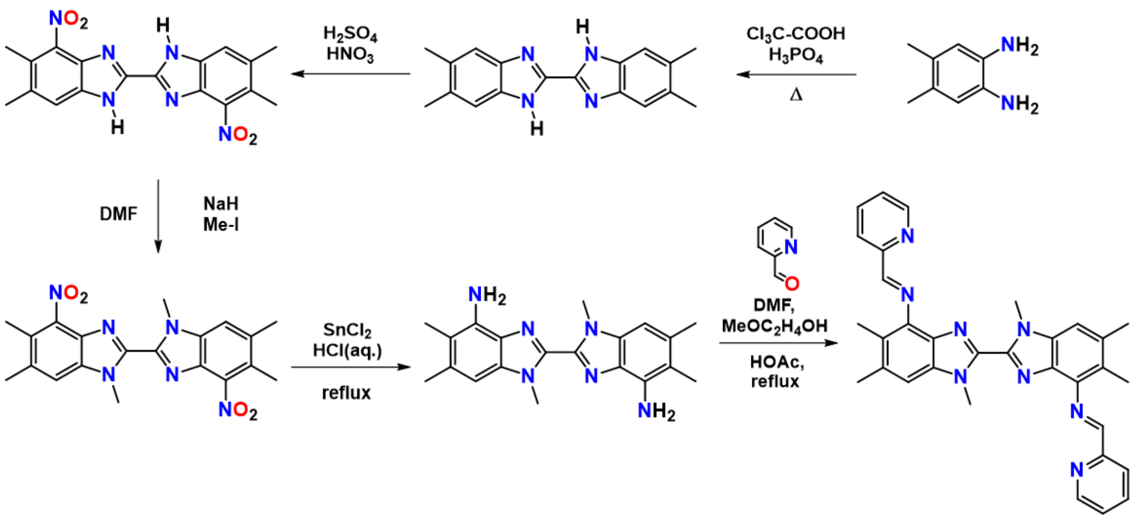

Figure S7: Synthesis route of the $^{\mathbf{Me}}$**bpbbi** ligand.

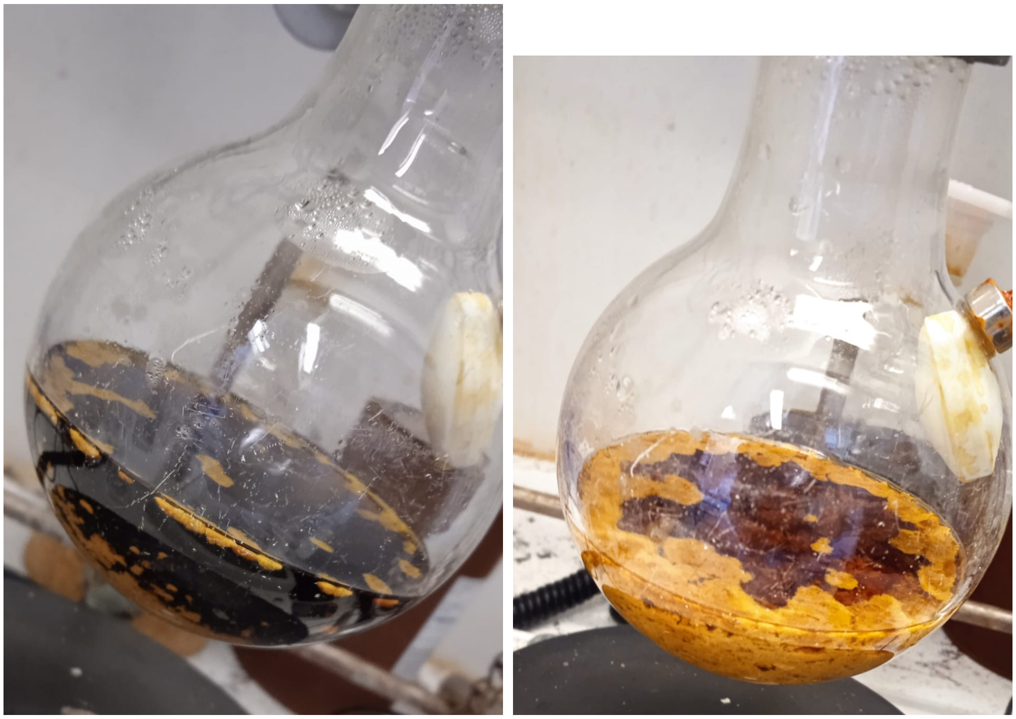

Figure S8: Voluminous crystallization of the ligand observed upon cooling down the DMF solution as reported by Reedijk et al.[S4] after the Schiff base condensation reaction.

Hydrogenation of ligand

Under a protective N_2 atmosphere $^{\mathbf{Me}}$**bpbbi** (100.0 mg, 0.18 mmol) was suspended in THF (10 mL). While stirring ar room temperature, a 1 M solution of $NaHBEt_3$ (360 μL, 0.36 mmol) was added dropwise. Within 10 minutes, the yellow suspension turned into a clear, bright-red solution. When methanol (1mL) was added, the red color disappeared immediately and a pale off-white precipitate was formed. The precipitate was filetered and washed with methanol (3x 1 mL), and the remaining solid dried *in vacuo*. $^{\mathbf{Me}}\mathbf{bpbbi-H_4}$ was collected as a fluffy off-white solid. Yield: 82.20 mg (79%).

^{1}H-NMR (CD_2Cl_2, ppm): 8.51 (d, 2H, py), 7.52 (dd, 2H, py), 7.33 (d, 2H, py), 7.08 (dd, 2H, py), 6.60 (s, 2H, bi), 5.31 (s, 4H, CH_2), 5.31 (s, 6H, N-CH_3), 4.5 – 3.0 (broad unstructured, 2H, NH), 4.00 (s, 6H, N-CH_3), 2.37 (s, 6H, bi-CH_3), 2.20 (s, 6H, bi-CH_3)

^{1}3C-NMR (CD_2Cl_2, ppm): 166.6, 161.1, 150.7, 149.6, 138.7, 136.8, 134.2, 123.5, 122.2, 122.1, 114.1, 100.7, 52.3, 32.7, 22.4, 13.6.

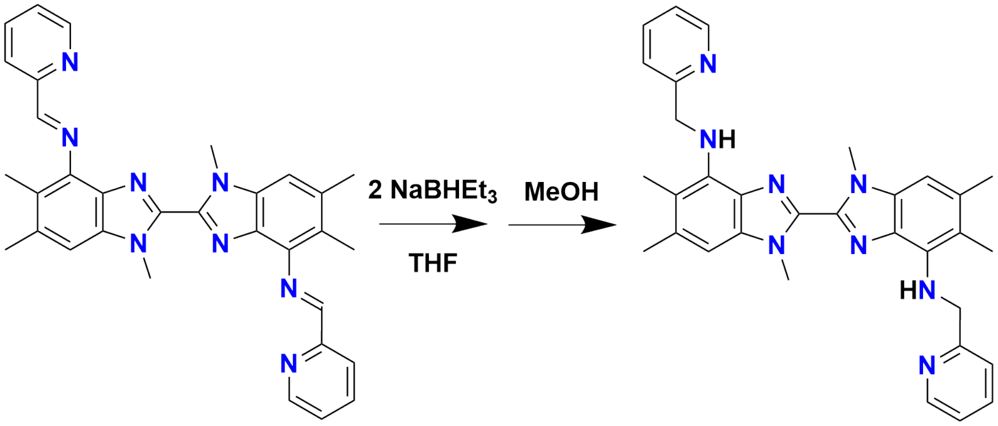

Figure S9: Reaction scheme for the hydrogenation of the ligand

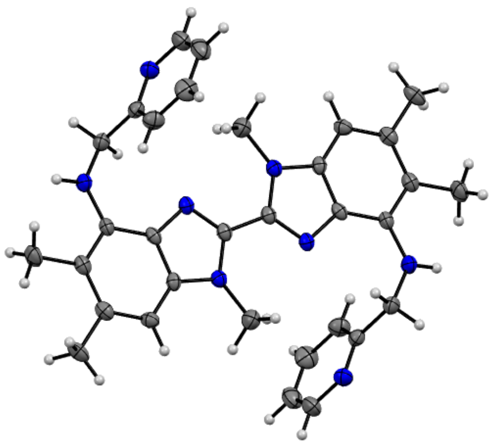

Figure S10: Crystal structure of $^{\mathbf{Me}}\mathbf{bpbbi-H_4}$.

Complex {Fe_2} synthesis

A 526.65 mg (1.00 mmol) sample of the ligand was suspended in THF and a sample of 253.50 g of FeCl2 (2.00 mmol) was added. The yellow suspension is stirred overnight and slowly evolves a green colour. The resulting green suspension is filtered and washed with diethyl ether to yield 763.50 mg of a pale green powder. Yield: 97.8%.

^{1}H-NMR (Methanol-d_4, 323 K, ppm. Values assigned to structure [Fe_2Cl_4]): 123.20 (1H, imine C-H), 51.72 (1H), 49.54 (1H), 16.81 (1H), 16.41 (1H), 8.32 (3H, methyl), 2.24 (1H), 0.97 (3H, methyl), 0.27 (3H, methyl).

Elemental analysis. Calculated for C36H38Cl4Fe2N8O([Fe_2Cl_4]·THF): C, 50.74%; H, 4.49%; N, 13.15%. Found: C, 50.22 ± 0.08%; H, 4.10 ± 0.18%; N, 13.30 ± 0.03%.

UV-vis (MeOH, 293 K, nm): 500-750 (broad, max. 651), 300-400 (intense). See the respective chapters below for more details and the spectra.

Crystal Preparations

[Fe_2Cl_4]·THF: 5-10 mg of an amorphous sample of freshly synthesized Fe2 were dissolved in 5-10 mL of dry methanol and filled into narrow 5 mL glass vials which were placed into a 20 mL glass vial with a screw cap. The 20 mL glass vial was then filled with 5-10 mL of dry THF and the vial closed and left at room temperature. Large green crystals deposited inside the narrow 5 mL vials within hours and were harvested after 2 days. [Fe_2Cl_4]· 2MeOH: 5-10 mg of an amorphous sample of freshly synthesized Fe2 were dissolved in 5-10 mL of dry methanol and filled into narrow 5 mL glass vials which were placed into a 20 mL glass vial with a screw cap. The 20 mL glass vial was then filled with 5-10 mL of dry toluene and the vial closed and left at room temperature. Deposition of crystals above the surface of methanol solutions was visible within a day. Crystals which had also formed on the bottom of the vials were harvested after a week. [Fe_2Cl_2]Cl_2: 5-10 mg of an amorphous sample of freshly synthesized Fe2 were dissolved in 1-2 mL of dry methanol in a 20 mL screw cap vial, layered with 1-2 mL of dry methanol and subsequently layered with 10-15 mL of dry

diethylether. The vial was then placed in the glovebox freezer at -40°C. Within a week, dark blue crystals deposited at the bottom of the vial which were harvested in a pre-cooled frit and washed with diethylether. $[Fe_2Cl_3]Cl$: 5-10 mg of an amorphous sample of freshly synthesized Fe2 were dissolved in 5-10 mL of dry methanol and filled into narrow 5 mL glass vials which were placed into a 20 mL glass vial with a screw cap. The 20 mL glass vial was then filled with 5-10 mL of dry diethylether and the vial closed placed in the glovebox freezer at -40°C. Within a week, dark blue crystals deposited at the bottom of the narrow 5 mL vials which were harvested in a pre-cooled frit and washed with diethylether.

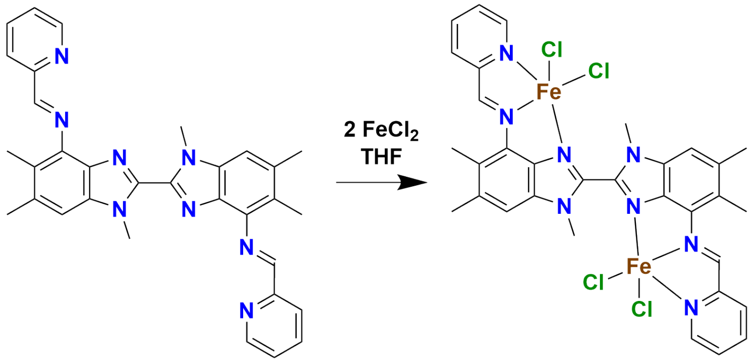

Figure S11: Synthesis route to metallation of the ^{Me}**bpbbi** ligand.

NMR

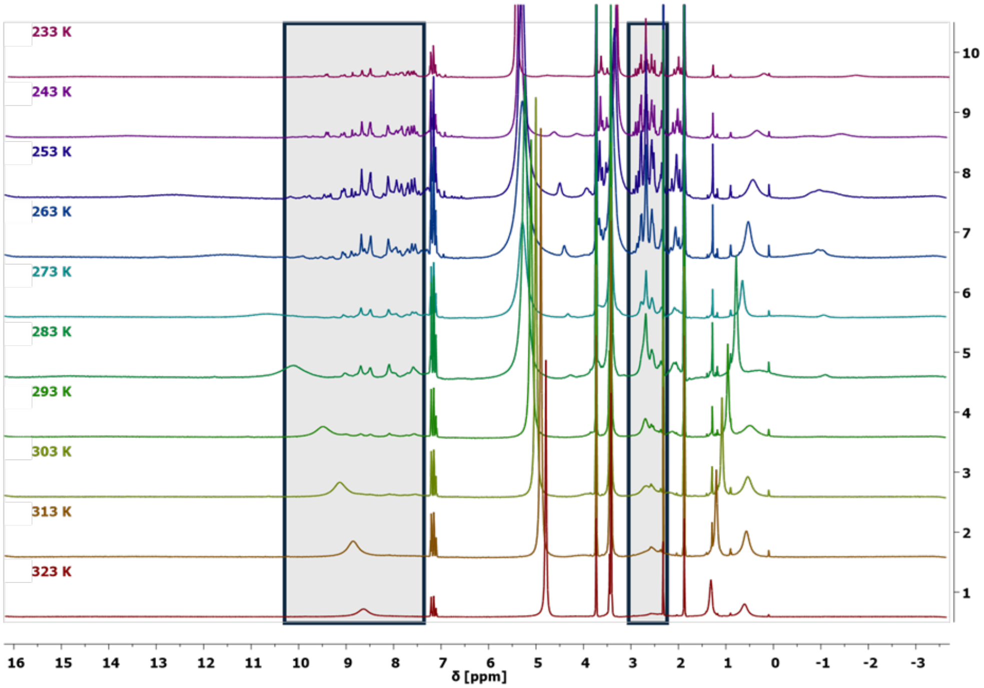

Figure S12: Variable temperature ^{1}H-NMR spectra (400 MHz) of {**Fe$_2$**} in methanol-d$_4$. The aromatic region is highlighted, indicating the poorly resolved signals at low temperature due to ligand exchange.

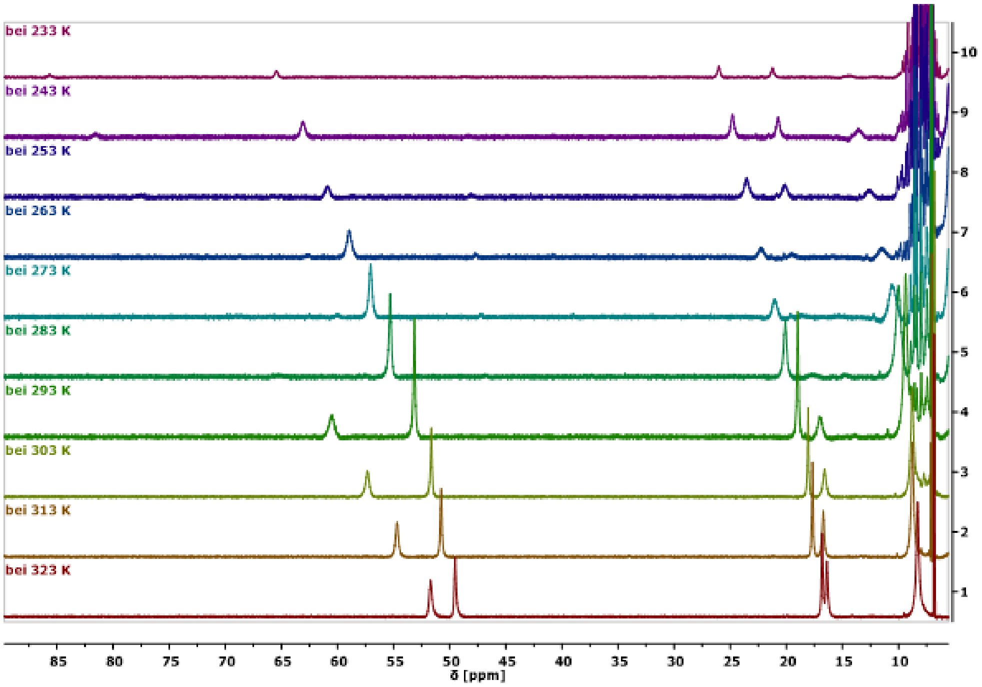

Figure S13: Variable temperature ^{1}H-NMR spectra (400 MHz) of {**Fe$_2$**} in methanol-d$_4$. Zoom in the region from 5 to 90 ppm.

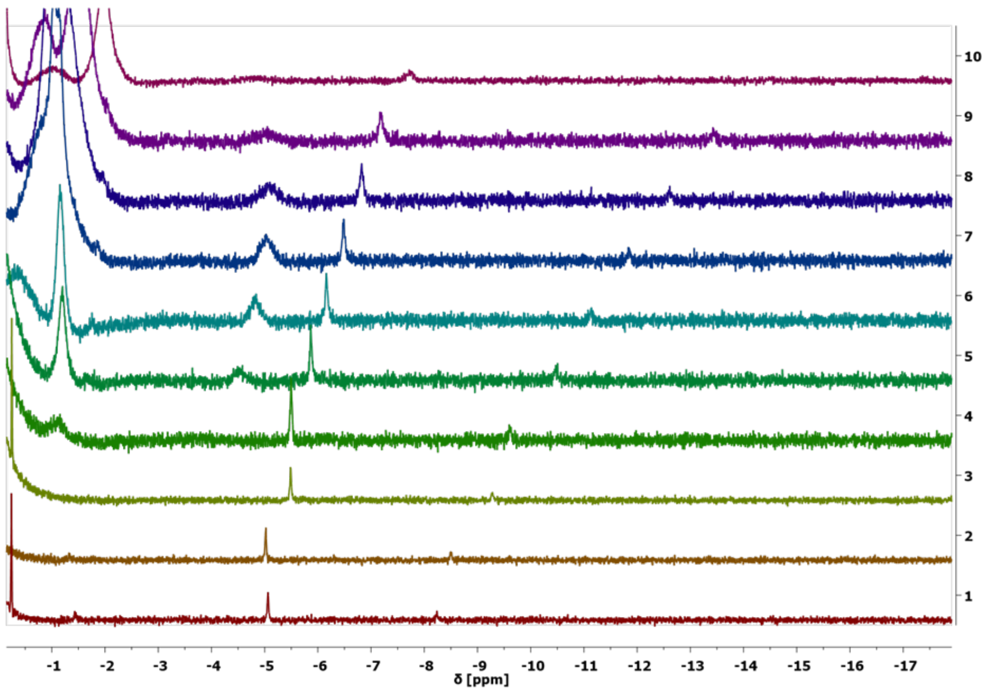

Figure S14: Variable temperature ^{1}H-NMR spectra (400 MHz) of {$\mathbf{Fe_2}$} in methanol-d_4. Zoom in the region from -18 to 0 ppm.

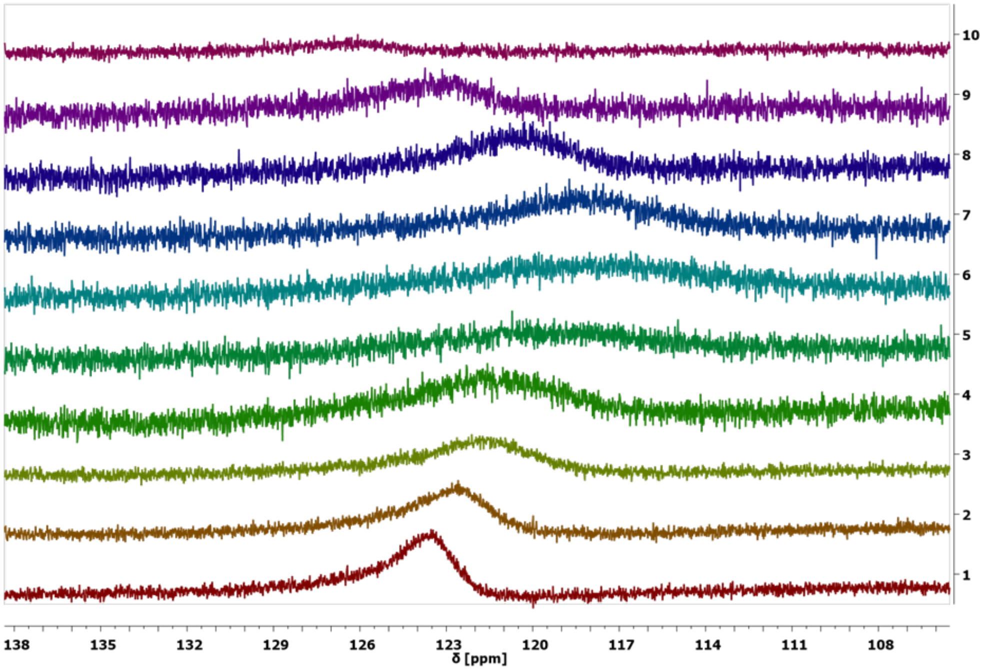

Figure S15: Variable temperature ^{1}H-NMR spectra (400 MHz) of $\{\mathbf{Fe_2}\}$ in methanol-d$_4$. Zoom in the region from 105 to 138 ppm.

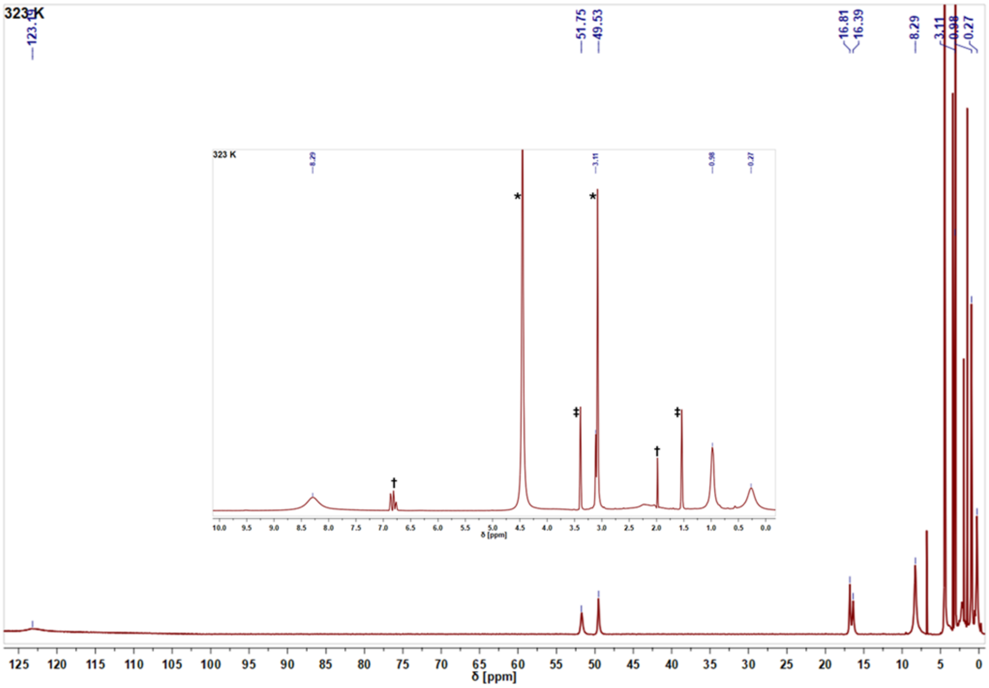

Figure S16: Full ^{1}H-NMR spectrum of $\{\mathbf{Fe_2}\}$, data collected at 50°C, inlay highlighting the resonances between 0-10 ppm; symbols indicate solvent residual signals, i.e., asterisks ($*$) indicating methanol, symbol ‡ indicating co-crystallized THF, symbol † indicating residual toluene.

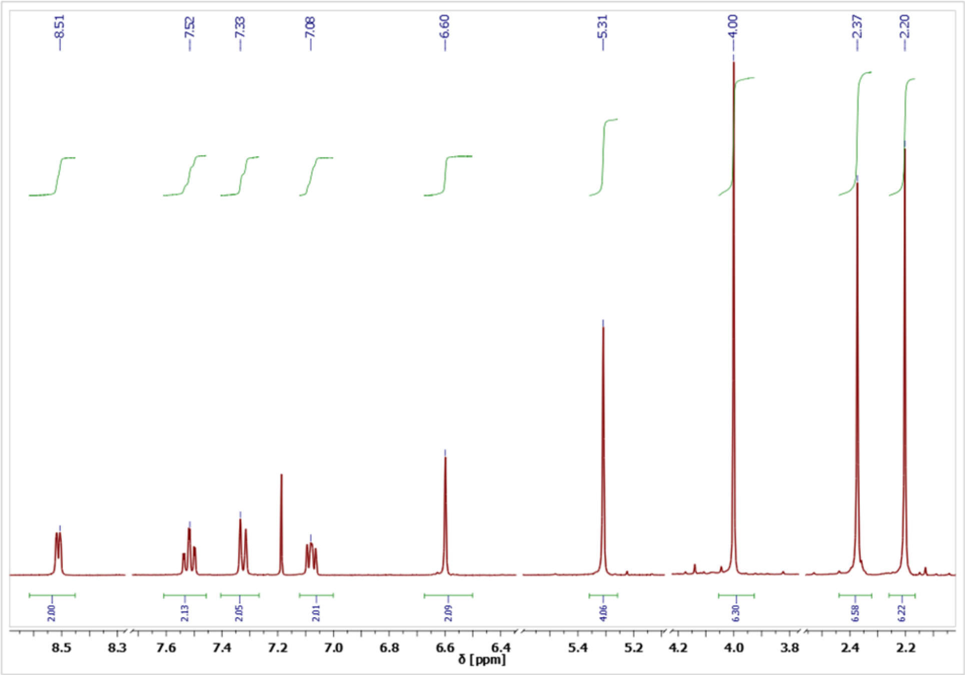

Figure S17: ^{1}H-NMR spectrum of $^{\mathbf{Me}}\mathbf{bpbbi-H_4}$ in CCl_2D_2.

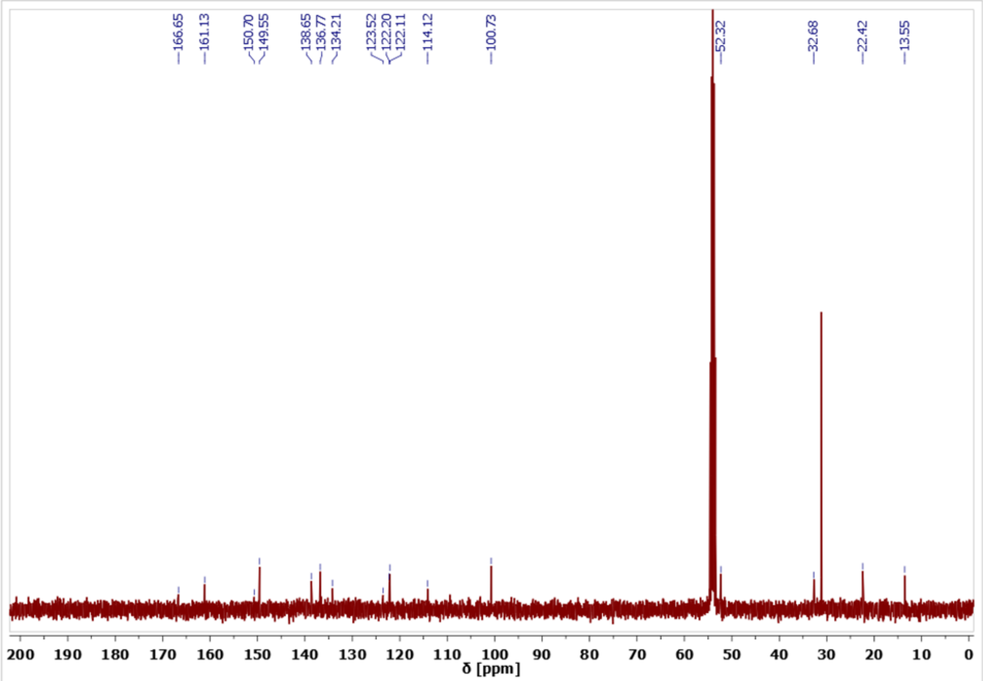

Figure S18: ^{13}C-NMR spectrum of $^{\mathbf{Me}}\mathbf{bpbbi-H_4}$ in CCl_2D_2.

Elemental Analysis

Chemical Formula: C32H30N8 Molecular Weight/gmol^{-1}: 526,648

Theoretical Values: 72.98 % C, 5.74 % H, 21.28 % N, - % S, Contains fluorine? ☐ Yes ☑ No

Figure S19: Theoretical values of elemental analysis of ligand.

Final Results:

average	71.72 % C	5.86 % H	19.87 % N	% S
Std. dev.	0.12	0.20	0.10	

20.12.2023

Date/Signature

Figure S20: Elemental analysis of ligand.

Chemical Formula: $C_{36}H_{38}Cl_4Fe_2N_8O$ Molecular Weight/gmol^{-1}: 852.24

Theoretical Values: 50.74 % C, 4.49 % H, 13.15 % N, % S, Contains fluorine? ☐ Yes ☒ No

Figure S21: Theoretical values of elemental analysis of complex.

Final Results:

average	50.22 % C	4.10 % H	13.30 % N	% S
Std. dev.	0.08	0.18	0.03	

11.09.2024

Date/Signature

Figure S22: Elemental analysis of complex.

UV/vis

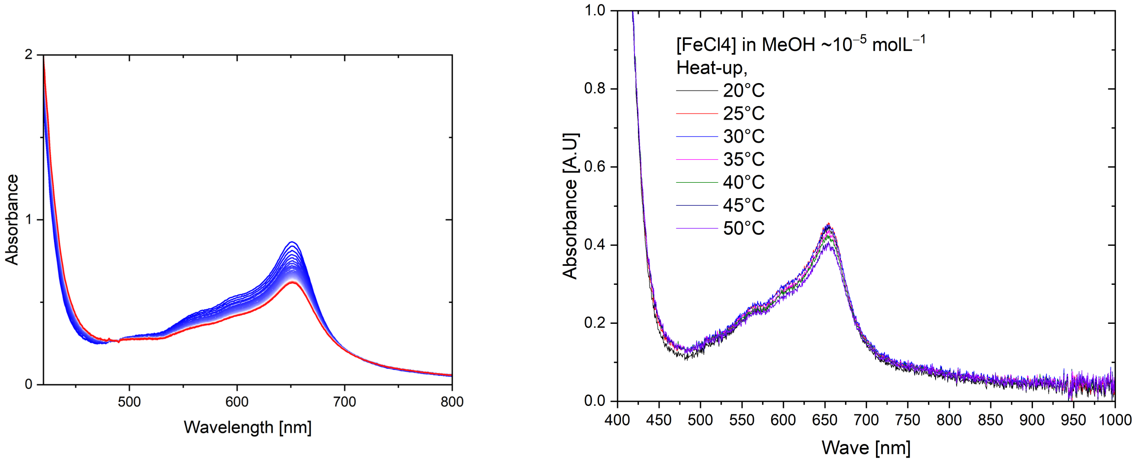

Figure S23: Variable temperature UV/vis spectra of {$\mathbf{Fe_2}$} complex from -40 °C to room temperature (**left** red line = RT) and from 20 °C to 50 °C (**right**).

IR Spectra

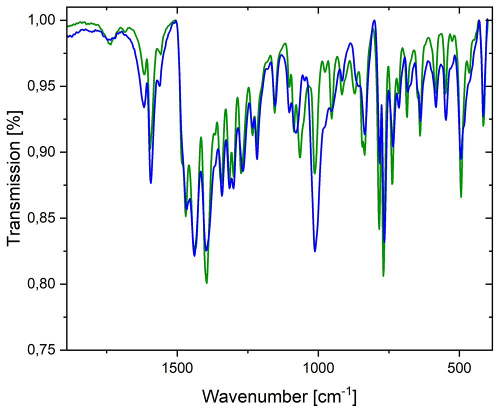

Figure S24: IR spectra of $\{\mathbf{Fe_2}\}$ complexes crystallized from methanol at -40°C (blue line) and room temperature (green line). Zoom in the region from 500 to 2000 cm^{-1}.

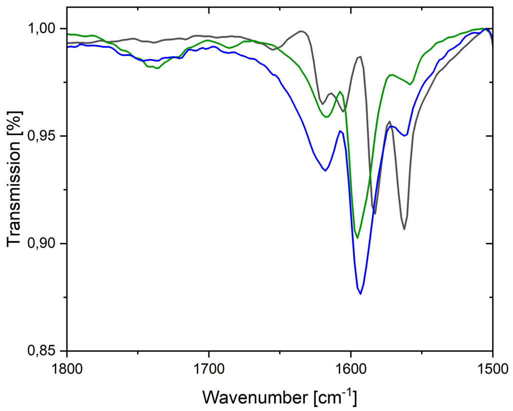

Figure S25: IR spectra of the ligand (black line) and $\{\mathbf{Fe_2}\}$ complexes crystallized from methanol at -40°C (blue line) and room temperature (green line). Zoom in the region from 1500 to 1800 cm^{-1}.

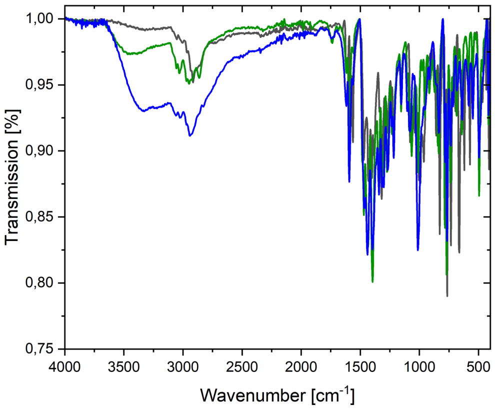

Figure S26: IR spectra of the ligand (black line) and {$\mathbf{Fe_2}$} complexes crystalized from methanol at -40°C (blue line) and room temperature (green line).

scXRD

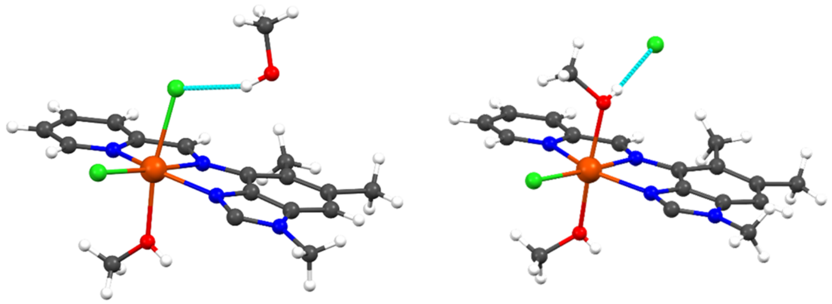

Figure S27: Structures of the two halves of the dinuclear complex {$\mathbf{Fe_2}$} from its respective solid-state structure denoted as {$\mathbf{Fe_2Cl_3}$}Cl, cut at the central C-C single bond; left: one chlorido ligand trans to methanol is only weakly bound to iron as indicated by an unusually long Fe-Cl bond, forming a hydrogen bond with one methanol solvate; right: two methanol molecules bound to iron as observed in the LT structure denoted as {$\mathbf{Fe_2Cl_3}$}Cl_2, with chloride bound to one coordinated methanol ligand through a hydrogen bond; side-by-side these two halves appear like a before-and-after of the ligand exchange process, trapped in crystallo.

Table S6: Crystal data and structure refinement for CCDC entry #2480870

Identification code	**Mebpbbi**
Empirical formula	$C_{32}H_{30}N_8$
Formula weight / g/mol	526.64
Temperature/K	150
Crystal system	triclinic
Space group	P-1
a/Å	10.5762(5)
b/Å	11.5648(5)
c/Å	13.3015(8)
α/°	115.622(2)
β/°	92.091(2)
γ/°	100.024(2)
Volume/Å^3	1433.06(13)
Z	2
ρ_{calc}/ g/cm^3	1.22
μ/mm^{-1}	0.076
F(000)	556
Crystal size/mm^3	0.10 × 0.10 × 0.10
Radiation	MoKα (λ = 0.71073)
2Θ range for data collection/°	3.936 to 55.038
Index ranges	$-13 \leq h \leq 13$, $-15 \leq k \leq 15$, $-17 \leq l \leq 17$
Reflections collected	25361
Independent reflections	6572 [R_{int} = 0.0518, R_{sigma} = 0.0500]
Data/restraints/parameters	6572/0/367
Goodness-of-fit on F^2	1.023
Final R indexes [I>=2σ (I)]	R_1 = 0.0642, wR_2 = 0.1730
Final R indexes [all data]	R_1 = 0.0786, wR_2 = 0.1832
Largest diff. peak/hole / e Å^3	0.39/-0.31

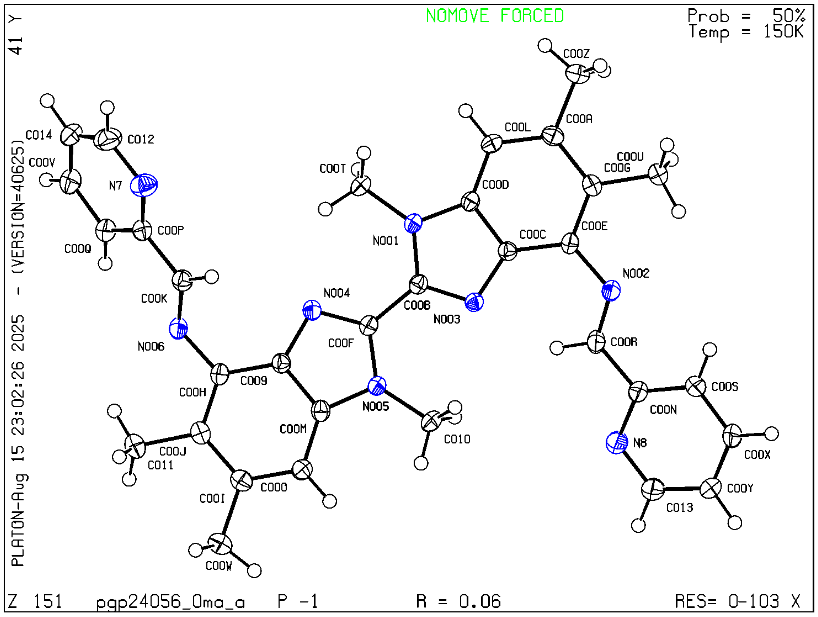

Figure S28: Structure fit of scXRD **CCDC entry 2480870**.

Table S7: Crystal data and structure refinement for CCDC entry #2480871

Identification code	**{Fe2Cl4} THF**
Empirical formula	$C_{36}H_{38}Cl_4Fe_2N_8O$
Formula weight/ g/mol	852.24
Temperature/K	150
Crystal system	monoclinic
Space group	$P2_1/c$
a/Å	16.713(2)
b/Å	15.8956(16)
c/Å	14.4748(15)
α/°	90
β/°	101.637(4)
γ/°	90
Volume/Å^3	3766.5(7)
Z	4
ρ_{calc}/ g/cm^3	1.503
μ/mm^{-1}	1.096
F(000)	1752
Crystal size/mm^3	0.10 × 0.10 × 0.10
Radiation	MoKα (λ = 0.71073)
2Θ range for data collection/°	3.572 to 52.786
Index ranges	-20 ≤ h ≤ 20, -19 ≤ k ≤ 19, -17 ≤ l ≤ 18
Reflections collected	40666
Independent reflections	7680 [R_{int} = 0.0798, R_{sigma} = 0.0601]
Data/restraints/parameters	7680/0/466
Goodness-of-fit on F^2	1.02
Final R indexes [I>=2σ (I)]	R_1 = 0.0666, wR_2 = 0.1739
Final R indexes [all data]	R_1 = 0.0898, wR_2 = 0.1949
Largest diff. peak/hole / e Å^3	0.63/-0.51

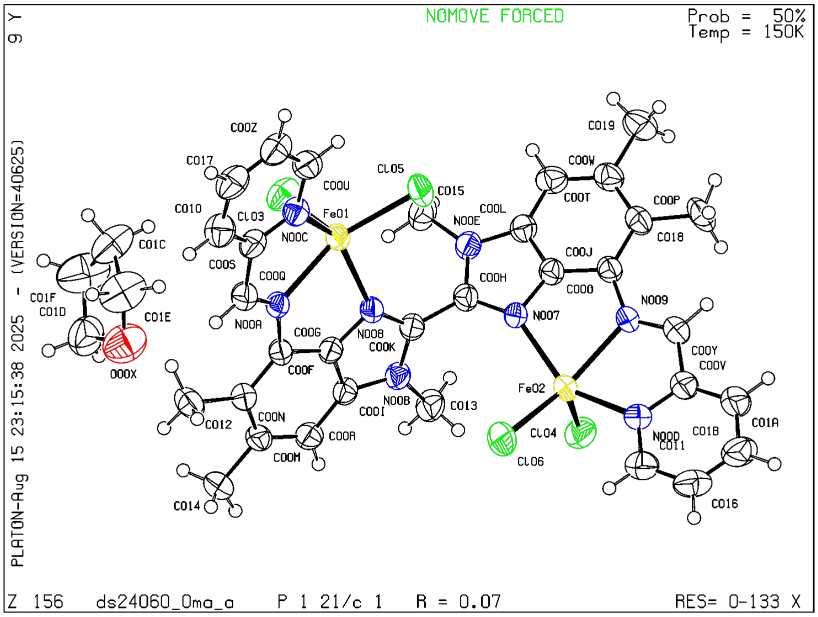

Figure S29: Structure fit of scXRD **CCDC entry 2480871**.

Table S8: Crystal data and structure refinement for CCDC entry #2480872

Identification code	**{Fe_2Cl_4}($MeOH$)$_2$**
Empirical formula	$C_{34}H_{38}Cl_4Fe_2N_8O_2$
Formula weight/ g/mol	860.24
Temperature/K	150.00(14)
Crystal system	triclinic
Space group	$P\bar{1}$
a/Å	11.5410(4)
b/Å	12.9420(4)
c/Å	14.1138(5)
α/°	74.433(3)
β/°	83.035(3)
γ/°	69.694(3)
Volume/Å^3	1903.65(12)
Z	2
ρ_{calc}/ g/cm^3	1.473
μ/mm^{-1}	9.06
F(000)	868
Crystal size/mm^3	0.265 × 0.147 × 0.133
Radiation	CuKα (λ = 1.54184)
2Θ range for data collection/°	7.5 to 145.92
Index ranges	-14 ≤ h ≤ 14, -14 ≤ k ≤ 16, -17 ≤ l ≤ 17
Reflections collected	28231
Independent reflections	7466 [R_{int} = 0.0502, R_{sigma} = 0.0371]
Data/restraints/parameters	7466/0/459
Goodness-of-fit on F^2	1.031
Final R indexes [I>=2σ (I)]	R_1 = 0.0416, wR_2 = 0.1125
Final R indexes [all data]	R_1 = 0.0445, wR_2 = 0.1156
Largest diff. peak/hole / e Å^3	0.86/-0.60

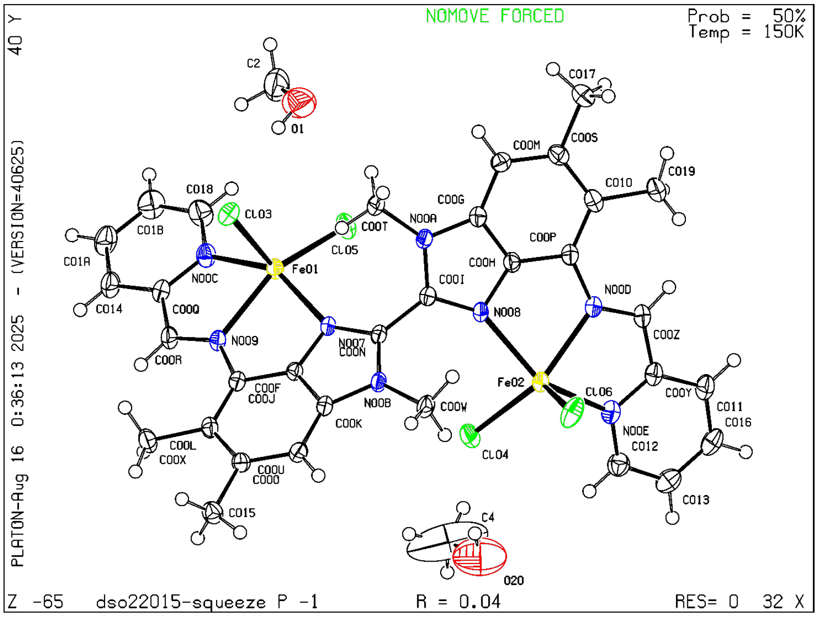

Figure S30: Structure fit of scXRD **CCDC entry 2480872**.

Table S9: Crystal data and structure refinement for CCDC entry #2480873

Identification code	**$\{Fe_2Cl_2\}Cl_2$**
Empirical formula	$C_{37}H_{50}Cl_4Fe_2N_8O_5$
Formula weight/ g/mol	940.35
Temperature/K	150
Crystal system	monoclinic
Space group	$C2/c$
a/Å	18.73(2)
b/Å	16.541(13)
c/Å	27.57(3)
α/°	90
β/°	106.25(5)
γ/°	90
Volume/Å^3	8203(14)
Z	8
ρ_{calc}/ g/cm^3	1.523
μ/mm^{-1}	1.021
F(000)	3904
Crystal size/mm^3	0.1 × 0.1 × 0.1
Radiation	MoKα (λ = 0.71073)
2Θ range for data collection/°	3.938 to 55.216
Index ranges	$-24 \leq h \leq 23$, $-21 \leq k \leq 21$, $-35 \leq l \leq 35$
Reflections collected	85547
Independent reflections	9491 [R_{int} = 0.0878, R_{sigma} = 0.0463]
Data/restraints/parameters	9491/0/529
Goodness-of-fit on F^2	1.036
Final R indexes [|>=2σ (I)]	R_1 = 0.0494, wR_2 = 0.1176
Final R indexes [all data]	R_1 = 0.0659, wR_2 = 0.1260
Largest diff. peak/hole / e Å^3	1.07/-0.63

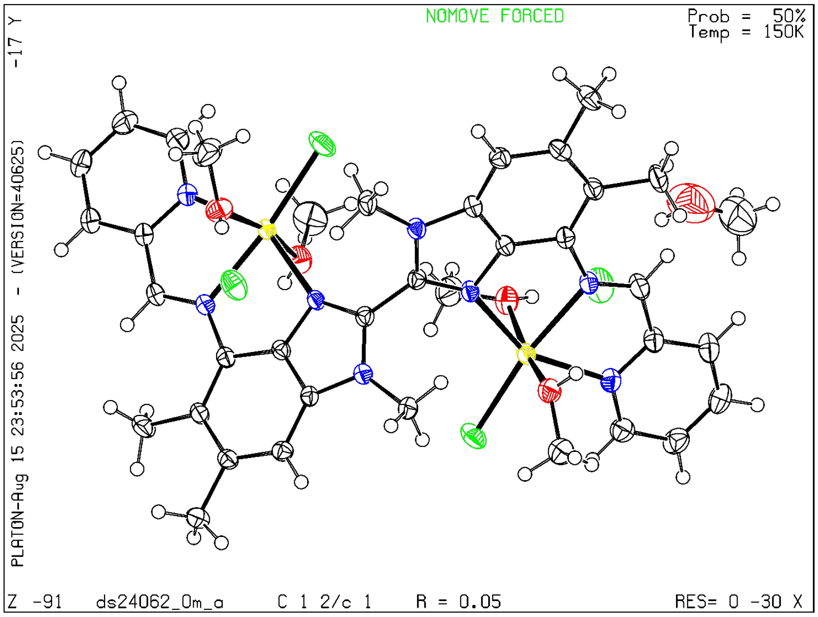

Figure S31: Structure fit of scXRD **CCDC entry 2480873**.

Table S10: Crystal data and structure refinement for CCDC entry #2480884

Identification code	**LT − {Fe_2Cl_3}Cl**
Empirical formula	$C37\,H_{50}Cl_4Fe_2N_8O_5$
Formula weight/ g/mol	940.35
Temperature/K	150
Crystal system	monoclinic
Space group	$P2_1/c$
a/Å	24.109(2)
b/Å	11.0622(11)
c/Å	17.2650(16)
α/°	90
β/°	106.526(3)
γ/°	90
Volume/Å^3	4414.4(7)
Z	4
ρ_{calc}/ g/cm^3	1.415
μ/mm^{-1}	0.949
F(000)	1952
Crystal size/mm^3	0.05 × 0.05 × 0.05
Radiation	MoKα (λ = 0.71073)
2Θ range for data collection/°	3.68 to 55.3
Index ranges	-31 ≤ h ≤ 31, -14 ≤ k ≤ 13, -22 ≤ l ≤ 22
Reflections collected	47994
Independent reflections	10178 [R_{int} = 0.0740, R_{sigma} = 0.0582]
Data/restraints/parameters	10178/0/524
Goodness-of-fit on F^2	1.069
Final R indexes [|>=2σ (I)]	R_1 = 0.0522, wR_2 = 0.1268
Final R indexes [all data]	R_1 = 0.0937, wR_2 = 0.1447
Largest diff. peak/hole / e Å^3	0.81/-0.35

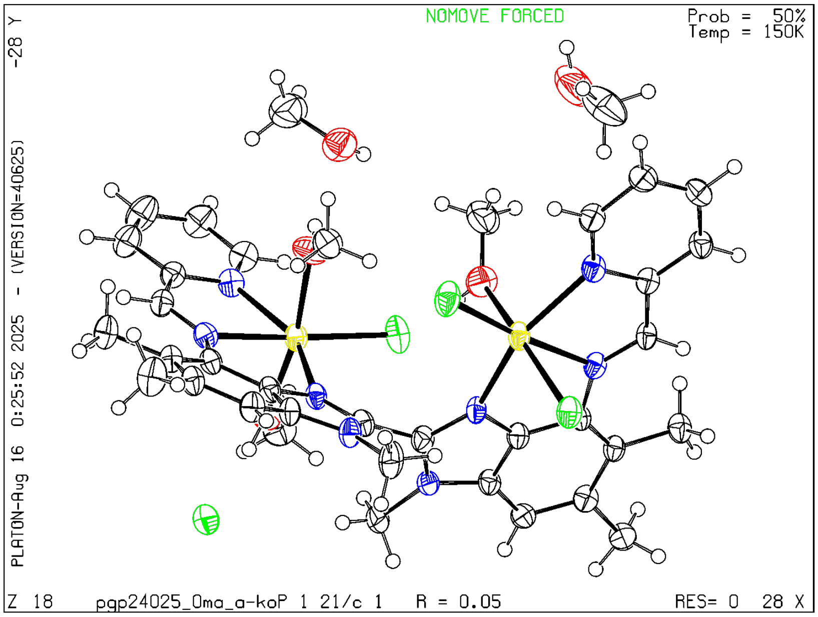

Figure S32: Structure fit of scXRD **CCDC entry 2480884**.

Table S11: Crystal data and structure refinement for CCDC entry #2526015

Identification code	$^{\mathbf{Me}}\mathbf{bpbbi-H_4}$
Empirical formula	$C32\,H_{34}N_8$
Formula weight/ g/mol	530.67
Temperature/K	150
Crystal system	monoclinic
Space group	$P2_1$/c
a/Å	8.0617(19)
b/Å	7.2667(16)
c/Å	22.943(6)
α/°	90
β/°	91.507(9)
γ/°	90
Volume/Å^3	1343.6(5)
Z	2
ρ_{calc}/ g/cm^3	1.312
μ/mm^{-1}	0.081
F(000)	564.0
Crystal size/mm^3	0.01 × 0.01 × 0.01
Radiation	MoKα (λ = 0.71073)
2Θ range for data collection/°	5.054 to 63.032 to 55.3
Index ranges	-11 ≤ h ≤ 11, -10 ≤ k ≤ 8, -33 ≤ l ≤ 33
Reflections collected	18474
Independent reflections	4230 [R_{int} = 0.1807, R_{sigma} = 0.1749]
Data/restraints/parameters	4230/0/184
Goodness-of-fit on F^2	1.028
Final R indexes [|>=2σ (I)]	R_1 = 0.0975, wR_2 = 0.1715
Final R indexes [all data]	R_1 = 0.1833, wR_2 = 0.2051
Largest diff. peak/hole / e Å^3	0.28/-0.33

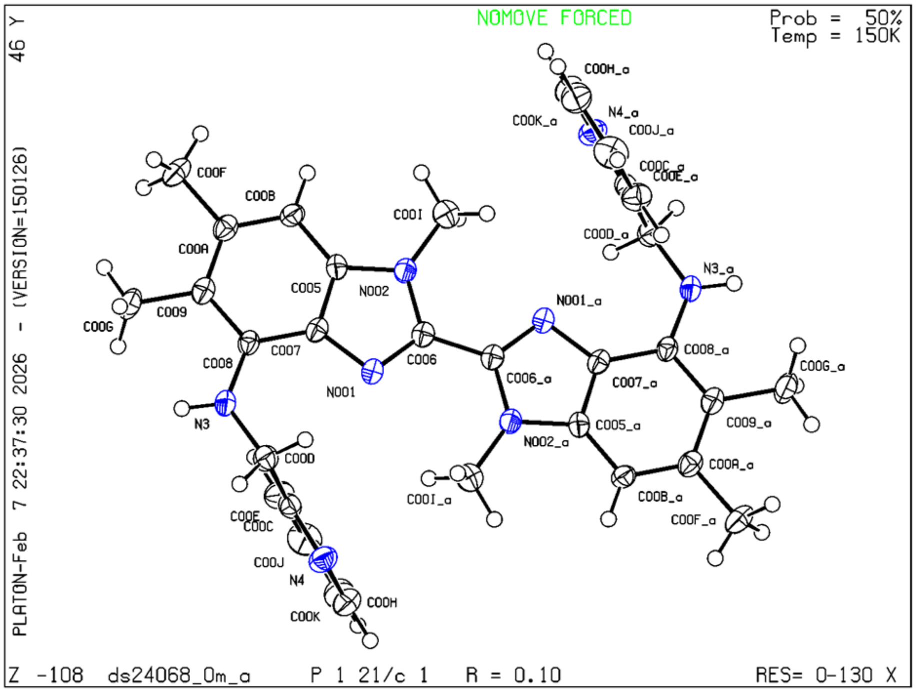

Figure S33: Structure fit of scXRD **CCDC entry 2526015**.

Magnetometry

Static susceptibility measurements. The static magnetic behaviour for both solid samples ([Fe_2Cl_4] and [Fe_2Cl_2]Cl_2) was investigated on polycrystalline samples in an MPMS-XL SQUID magnetometer from 2 to 250 K. Magnetization as a function of applied field was investigated in the field and temperature range of 0–5 T and 2–5 K, respectively. The data were corrected for diamagnetic contributions. The magnetic susceptibility data and magnetization data were fit to the following spin Hamiltonian:

$$\widehat{\mathrm{H}} = g_{\mathrm{iso}}\mu_{\mathrm{B}}\vec{H}\sum_{i=1}^{2}\widehat{\mathrm{S}}_i \; - \; J\left(\widehat{\mathrm{S}}_1 \cdot \widehat{\mathrm{S}}_2\right) \; + \; D\left[\widehat{\mathrm{S}}_z^2 - \frac{1}{3}S(S+1)\right] \; + \; E\left(\widehat{\mathrm{S}}_x^2 - \widehat{\mathrm{S}}_y^2\right) \qquad (1)$$

Here, g_{iso} is an isotropic g value (assumed equal for both iron atoms to avoid overparametrisation), μ_{B} is the Bohr magneton, $\vec{H}$ is the applied magnetic field, J is the isotropic superexchange coupling constant, D is the axial zero-field splitting and E is the rhombic zero-field-splitting (assumed equal for both iron atoms to avoid overparametrisation). $\widehat{\mathrm{S}}_i$ are the spin operators. Parallel least squares fitting in a full-matrix diagonalisation approach of both, DC-susceptibility and magnetisation data was carried out using the MagProp module of "DAVE" program package [cite: Philip Tregenna-Piggott et al., Data Analysis and Visualization Environment version 2.2, 2013] in a weighted iterative manner. This means that, alternatingly, both datasets were fitted using the parameters from the prior fit of the other dataset while fixing the parameters the respective data is less sensitive to and varying the others (DC-susceptibility varied parameters: g_{iso}, J; magnetisation varied parameters: D, E). This routine yielded the parameters given in Table S12.

Table S12: Parameters obtained from the fit of the experimental magnetic data to the Hamiltonian given in equation 1. For further details see the text above. R_χ and R_M are the χ^2 residuals from the corresponding fits of both data sets.

	g_{iso}	J/cm^{-1}	D/cm^{-1}	E/cm^{-1}	R_χ	R_M
[Fe_2Cl_4]	2.12	−0.05	−10.09	−3.35	0.1401	0.5033
[Fe_2Cl_2]Cl_2	2.059	−0.03	−5.49	−0.06	0.2784	2.6621

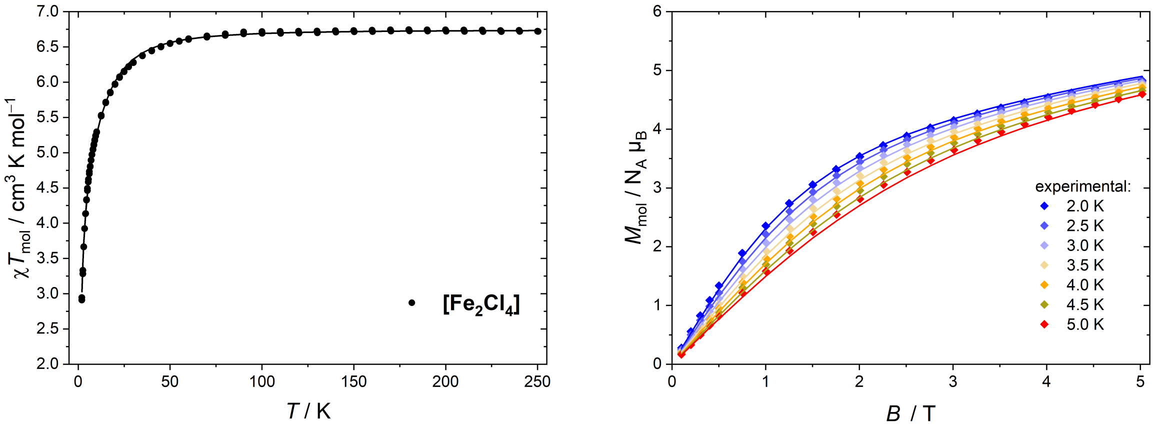

Figure S34: Experimental temperature dependent magnetic susceptibility with an applied static magnetic field of 0.2 T (left) and magnetisation curves (right) for[Fe_2Cl_4] each drawn together with the corresponding fits (lines) yielding the parameters given in Table S12 and the spin Hamiltonian in equation 1. Further details are explained in the text above.

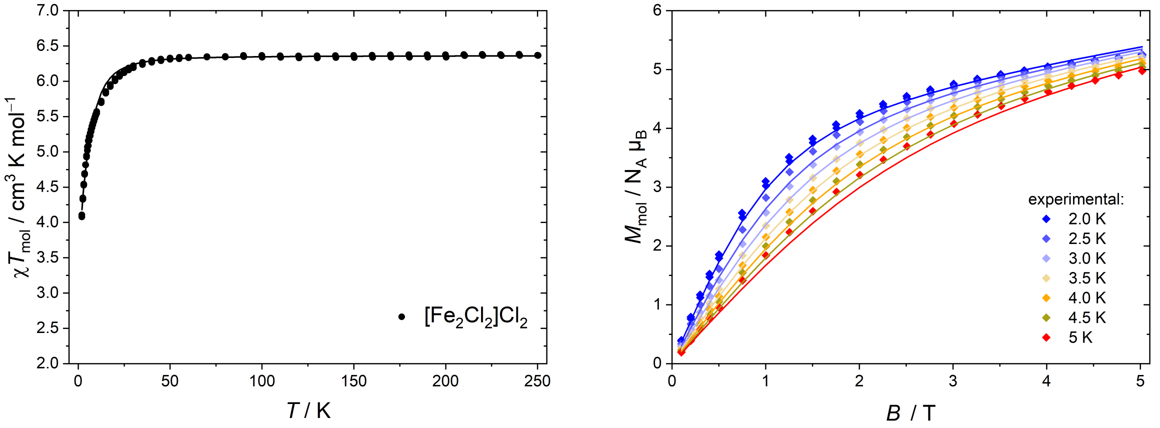

Figure S35: Experimental temperature dependent magnetic susceptibility with an applied static magnetic field of 0.2 T (left) and magnetisation curves (right) for[Fe_2Cl_4] each drawn together with the corresponding fits (lines) yielding the parameters given in Table S12 and the spin Hamiltonian in equation 1. Further details are explained in the text above.

Electrochemical Measurements

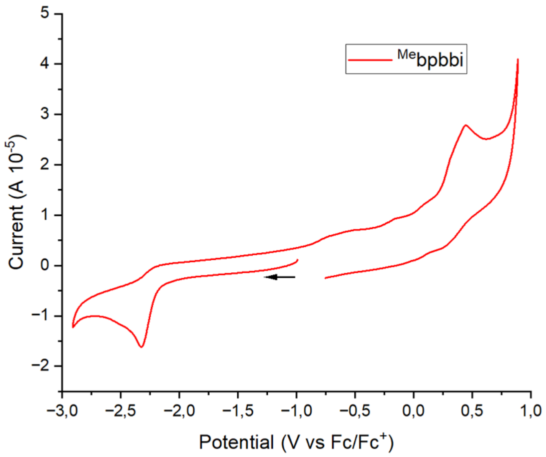

Figure S36: CV of ligand 1 mM with 0.2 M $TBAPF_6$ dissolved in 10 mL DMF at 100 mV/s.

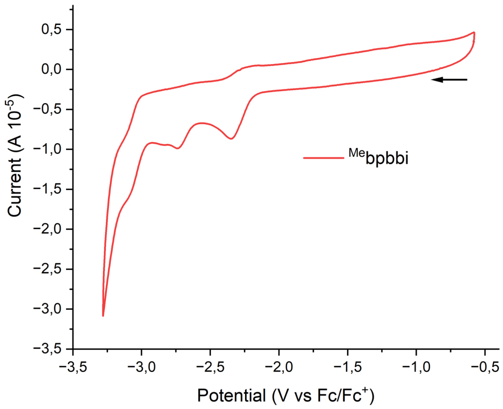

Figure S37: CV of ligand 1 mM with 0.2 M $TBAPF_6$ dissolved in 10 mL DMF at 100 mV/s.

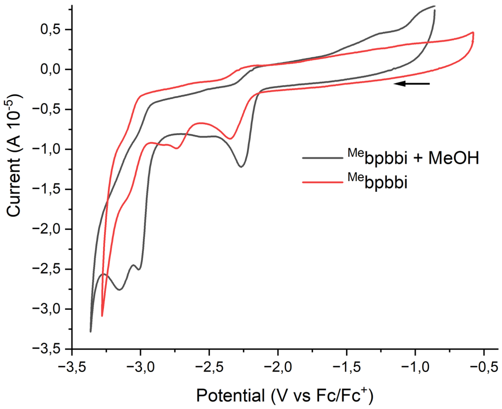

Figure S38: Comparison of CV's of ligand 1 mM with 0.2 M $TBAPF_6$ dissolved in 10 mL DMF (red line) and after the addition of a drop of methanol (black line) at 100 mV/s.

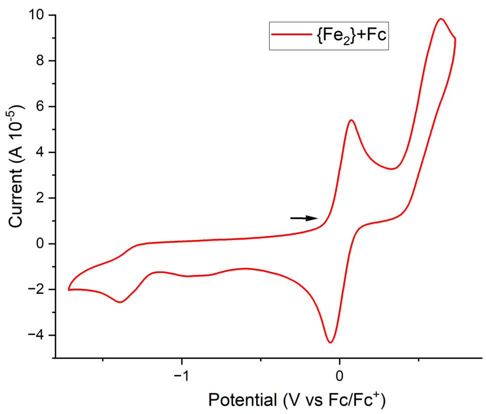

Figure S39: CV of $\{\mathbf{Fe_2}\}$ 1 mM with 1mM ferrocene and 0.1 M LiCl dissolved in 10 mL methanol at 100 mV/s.

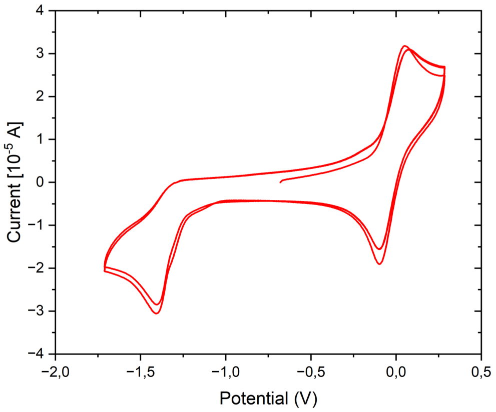

Figure S40: CV of {**Fe$_2$**} 1 mM with 1mM ferrocene and 0.1 M LiCl dissolved in 10 mL methanol at 100 mV/s.

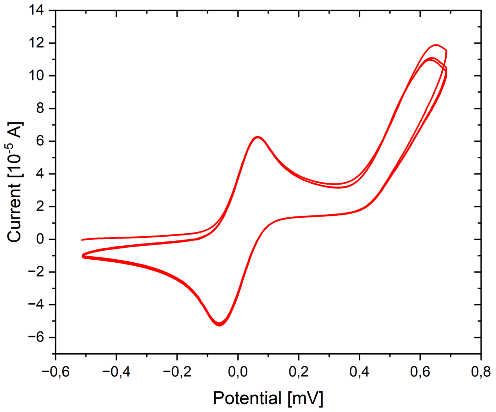

Figure S41: CV of {**Fe_2**} 1 mM with 1mM ferrocene and 0.1 M LiCl dissolved in 10 mL methanol at 100 mV/s.

References

(S1) Sheldrick, G. M. A short history of *SHELX*. *Acta Crystallographica Section A* **2008**, *64*, 112–122.

(S2) Sheldrick, G. M. Crystal structure refinement with *SHELXL*. *Acta Crystallographica Section C* **2015**, *71*, 3–8.

(S3) Macrae, C. F.; Edgington, P. R.; McCabe, P.; Pidcock, E.; Shields, G. P.; Taylor, R.; Towler, M.; van de Streek, J. *Mercury*: visualization and analysis of crystal structures. *Journal of Applied Crystallography* **2006**, *39*, 453–457.

(S4) Muller, E.; Bernardinelli, G.; Reedijk, J. 4,4'-Bis(2-Picolinimino)-2,2'-Bibenzimidazoles: A New Class of Dinucleating Ligands Which Allow for a Tuning of the Metal-Metal Distance. Structures and Properties of a Dicopper(II) Complex and of Two Oxygenation Products of a Dicopper(I) Complex; a Tentative Coordination Chemical Modeling of Hemocyanin. *Inorg. Chem.* **1995**, *34*, 5979–5988.
